# Effects of End-Caps on the Atropisomerization, Polymerization, and the Thermal Properties of *ortho*-Imide Functional Benzoxazines

**DOI:** 10.3390/polym11030399

**Published:** 2019-03-01

**Authors:** Kan Zhang, Yuqi Liu, Zhikun Shang, Corey J. Evans, Shengfu Yang

**Affiliations:** 1 Research School of Polymeric Materials, School of Materials Science and Engineering, Jiangsu University, Zhenjiang 212013, China; liuyuyu1196009474@163.com (Y.L.); 18852854312@163.com (Z.S.); 2 Department of Chemistry, University of Leicester, Leicester LE1 7RH, UK; cje8@leicester.ac.uk

**Keywords:** benzoxazine, *ortho*-imide, atropisomerization, thermal properties

## Abstract

A new type of atropisomerism has recently been discovered in 1,3-benzoxazines, where the intramolecular repulsion between negatively charged oxygen atoms on the imide and the oxazine ring hinders the rotation about the C–N bond. The imide group offers a high degree of flexibility for functionalization, allowing a variety of functional groups to be attached, and producing different types of end-caps. In this work, the effects of end-caps on the atropisomerism, thermally activated polymerization of *ortho*-imide functional benzoxazines, and the associated properties of polybenzoxazines have been systematically investigated. Several end-caps, with different electronic characteristics and rigidities, were designed. ^1^H and ^13^C nuclear magnetic resonance (NMR) spectroscopy and density functional theory (DFT) calculations were employed to obtain structural information, and differential scanning calorimetry (DSC) and in situ Fourier transform infrared (FT-IR) spectroscopy were also performed to study the thermally activated polymerization process of benzoxazine monomers. We demonstrated that the atropisomerization can be switched on/off by the manipulation of the steric structure of the end-caps, and polymerization behaviors can be well-controlled by the electronic properties of the end-caps. Moreover, a trade-off effect were found between the thermal properties and the rigidity of the end-caps in polybenzoxazines.

## 1. Introduction

Aromatic polymers have attracted much attention in the fields of microelectronics, aerospace, and defense technologies, due to their excellent electrical, mechanical, and thermal properties [1]. Amongst the different types of polymers, polyimides (PIs) are one of the most well-known engineering plastics, with a high level of thermal stability, high chemical resistance, and excellent mechanical properties originating from their rigid molecular structures and strong intermolecular interactions [2]. However, highly aromatic PIs generally have poor processability and solubility, due to inherent macromolecular stiffness and strong inter-chain forces; hence, they do not always meet requirements for specialty applications. To improve the processability of PIs, various approaches have been developed, e.g., by introducing flexible linkages or bulky substituents into the polymeric chains [3,4,5].

Since an early report by Holly and Cope in 1944 [6], 1,3-benzoxazine has been widely investigated and reported upon, due to its outstanding properties [7,8,9]. Polybenzoxazines, which can be readily obtained by the polymerization of 1,3-benzoxazines, possess a set of highly desirable properties, such as very high chemical and thermal resistance [10,11], excellent physical and mechanical properties [12], low flammability [13], low dielectric constants and dissipation factors [14,15,16], and low surface free energy [17,18,19]. The most remarkable and unique characteristics of polybenzoxazines are their extraordinarily rich molecular design flexibilities that allow for the construction of a variety of structures with tailored properties. 

Polyimide/benzoxazine blends and imide-functional benzoxazines have been extensively studied in recent years [20,21,22,23,24]. Interestingly, imide-functional benzoxazines with *ortho*-substituents have been shown to be superior to their *para*-substituted counterparts. For example, *ortho*-phthalimide functional benzoxazines showed great advantage in the synthesis of benzoxazine monomers, such as a shorter reaction time, higher yield, and ease of purification, when compared with *para*-phthalimide functional benzoxazines. Besides, the subsequent thermal conversion of *ortho*-phthalimide-functional polybenzoxazines into polybenzoxazoles (PBOs) provides an environmentally friendly alternative for preparing of PBO-related products [21]. In addition, *ortho*-imide benzoxazines like *ortho*-norbornene-containing benzoxazine monomers can further extend the advantages of smart *ortho*-imide benzoxazines [25,26]. On the other hand, this class of *ortho*-imide functional benzoxazines may also exhibit atropisomerization, which is unexpected, and has been discovered only recently [27]. The atropisomerism results from the intramolecular repulsion between the oxygen atoms in the tetrahydrophthalimide and oxazine ring of *ortho*-tetrahydrophthalimide functional benzoxazines, which was supported by density functional theory (DFT) calculations [27].

**Scheme 1:**
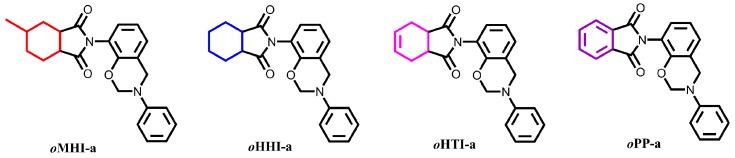
Structures of *ortho*-imide functional benzoxazines with various end-caps in this study.

This work was dedicated towards systematically studying how the electronic properties and rigidities of end-caps affect the atropisomerization and polymerization behaviors of *ortho*-imide functional benzoxazine monomers. Four types of benzoxazine monomers were designed, with the functionalities of 4-methyl-hexahydrophthalimide, hexahydrophthalimide, tetrahydrophthalimide, and phthalimide in the *ortho* position, with respect to the oxygen group in the oxazine ring of the benzoxazine (Scheme 1). These end-caps were chosen because of the diverse steric structures and their well-known electronegativities and rigidities. Through a combination of experimental measurements, including ^1^H and ^13^C nuclear magnetic resonance (NMR) spectroscopy, Fourier transform infrared (FT-IR) spectroscopy, differential scanning calorimetry (DSC), and DFT calculations, we intended to demonstrate how the atropisomerisms of the monomers can be manipulated, how the end-caps influence thermally activated polymerization, and the properties of the resulting polybenzoxazines. Ultimately, we hope that this work can establish the foundations for the design of new thermosets based on polybenzoxazines with controlled properties.

## 2. Experimental Section

### 2.1. Materials

*o*-Aminophenol (>98%), hexahydro-4-methylphthalic anhydride (98%), *cis*-1,2-cyclohexanedicarboxylic anhydride (99%), *cis*-1,2,3,6-tetrahydrophthalic anhydride (98%), phthalic anhydride (98%), and paraformaldehyde (99%) were used as received from Energy-Chemical. Aniline was purchased from Aladdin Reagent Co., Ltd., Shanghai, China, and it was purified by distillation. Acetic acid, hexanes, and xylenes were supported by Shanghai First Chemical Co., and were used as received. 2-(3-Phenyl-3,4-dihydro-2H-benzo[e][1,3]oxazin-8-yl)-3a,4,7,7a-tetrahydro-1H-isoindole-1,3(2H)-dione (abbreviated as *o*HTI-a), was synthesized using the procedure described in our recent work [27]. 

### 2.2. Characterization 

^1^H NMR and ^13^C NMR spectra were recorded with an NMR spectrometer (Bruker AVANCE II, 400 MHz, Bruker, Fällanden, Switzerland) in CDCl_3_/DMSO-*d_6_* using tetramethylsilane (TMS) as the internal standard. The average number of transients for ^1^H and ^13^C NMR measurements were 64 and 1024, respectively. Two-dimensional ^1^H–^13^C heteronuclear multiple quantum coherence (HMQC) was also examined. A Nicolet Nexus 670 Fourier transform infrared (FT-IR, Nicolet, Madison, WI, USA) spectrophotometer was used to obtain FT-IR spectra, the system was equipped with a deuterated triglycine sulfate (DTGS) detector and a dry air purge unit. The spectra were recorded with 32 co-added scans at a spectral resolution of 4 cm^−1^ in the frequency range of 400–4000 cm^−1^. All of the benzoxazine monomers synthesized were finely ground with potassium bromide powder and pressed into a disk, and the spectrum was taken in transmission mode. Elemental analysis was performed by an elementary analyzer (Elementar Vario EL-III, Hanau, Germany). DSC was performed by the use of NETZSCH DSC model 204f1 (NETZSCH, Selb, Germany). In tests for DSC, the heating rate was 10 °C/ min, and the nitrogen flow rate was set as 60 mL/min, to ensure a nitrogen atmosphere. The thermal decomposition temperatures, the char yields of the benzoxazine monomers, and the corresponding polybenzoxazines were measured using a NETZSCH STA449-C Thermogravimetric Analyzer (NETZSCH, Selb, Germany). Thermogravimetric analysis (TGA) was performed from room temperature (RT) to 800 °C at a heating rate of 10 °C/min, with a flow rate of 40 mL/min in a nitrogen atmosphere.

### 2.3. Computational Methods

Computational chemistry calculations were carried out by using both the Gaussian03 and CASTEP suite of programs. For the G03 calculations, the B3LYP hybrid function was employed using a 6-31+G(d) basis set, with tight self-consistent field (SCF) convergence and an ultrafine pruning grid [28]. Structures were then optimized, and the harmonic vibrational frequencies were calculated, to show that a true structural minima had been found. 

### 2.4. Synthesis of 2-(2-Hydroxyphenyl)-5-methylhexahydro-1H-isoindole-1,3(2H)-dione (Abbreviated as oMHI)

Hexahydro-4-methylphthalic anhydride (7.41 g, 0.05 mol), *o*-aminophenol (5.41 g, 0.05 mol), and 80 mL of acetic acid were added into a 250 mL round flask, which was mixed thoroughly and heated under refluxed for 6 hr. After cooling to room temperature, the precipitate was filtered and washed with 500 mL of deionized water. The removal of water by evaporation afforded a white powder, which was recrystallized from isopropanol (yield ca. 79%). ^1^H NMR (400 MHz, DMSO-d_6_), ppm: *δ* = 1.71 (d, 3H, -CH_3_), 2.16–2.41 (m, 6H, -CH_2_-), 2.30 (m, 1H), 3.23–3.29 (d, 2H), 6.80–7.25 (4H, Ar), 10.40 (s, 1H, -OH). IR spectra (KBr), cm^−1^: 3232 (O-H stretching), 1767, 1682 (imide I), 1415 (imide II, C-N stretching), 763 (C=O bending). Anal. calcd. for C_15_H_17_NO_3_: C, 69.48; H, 6.61; N, 5.40. Found: C, 69.39; H, 6.63; N, 5.37.

### 2.5. Synthesis of 5-Methyl-2-(3-phenyl-3,4-dihydro-2H-benzo[e][1,3]oxazin-8-yl)hexahydro-1H-isoindole-1,3(2H)-dione (Abbreviated as oMHI-a)

Aniline (0.93 g, 0.01 mol), *o*MHI (2.59 g, 0.01 mol), paraformaldehyde (0.61 g, 0.02 mol), and 30 mL of xylenes were placed into a 100 mL round-bottom flask equipped with a reflux condenser. The mixture was stirred at 120 °C for 6 hr. When the reaction was complete, the mixture was cooled to room temperature to afford a crude product, which was recrystallized from toluene/acetone mixtures (1:1) (yield ca. 87%). ^1^H NMR (CDCl_3_), ppm: *δ* = 1.01 (m, 3H, -CH_3_), 1.28–2.76 (m, 6H, -CH_2_-), 2.23 (m, 1H), 2.94–3.23 (m, 2H), 4.65 (d, 2H, Ar-CH_2_-N, oxazine), 5.35 (d, 2H, O-CH_2_-N, oxazine), 6.92–7.34 (8H, Ar). IR spectra (KBr), cm^−1^: 1778, 1710 (imide I), 1498 (stretching of a trisubstituted benzene ring), 1376 (imide II), 1232 (C-O-C asymmetric stretching), 950 (oxazine ring related mode). Anal. calcd. for C_23_H_24_N_2_O_3_: C, 73.38; H, 6.43; N, 7.44. Found: C, 73.30%; H, 6.46%; N, 7.41%.

### 2.6. Synthesis of 2-(2-Hydroxyphenyl)hexahydro-1H-isoindole-1,3(2H)-dione (Abbreviated as oHHI)

*cis*-1,2-Cyclohexanedicarboxylic anhydride (7.41 g, 0.05 mol), *o*-aminophenol (5.41 g, 0.05 mol), and 80 mL of acetic acid were added into a 250 mL round flask, which was mixed thoroughly and heated under refluxed for 6 hr. After cooling to room temperature, the precipitate was filtered and washed with 500 mL of deionized water. Removal of water by evaporation afforded a pink powder. The resulting white product was recrystallized from isopropanol (yield ca. 83%). ^1^H NMR (400 MHz, DMSO-d_6_), ppm: *δ* = 1.42–1.81 (m, 8H, -CH_2_-), 3.10 (t, 2H), 6.88–7.30 (4H, Ar), 9.84 (s, 1H, -OH). IR spectra (KBr), cm^−1^: 3244 (O-H stretching), 1772, 1689 (imide I), 1406 (imide II, C-N stretching), 753 (C=O bending). Anal. calcd. for C_14_H_15_NO_3_: C, 68.56; H, 6.16; N, 5.71. Found: C, 68.48; H, 6.20; N, 5.69.

### 2.7. Synthesis of 2-(3-Phenyl-3,4-dihydro-2H-benzo[e][1,3]oxazin-8-yl)hexahydro-1H-isoindole-1,3(2H)-dione (Abbreviated as oHHI-a)

Aniline (0.93 g, 0.01 mol), *o*HHI (2.45 g, 0.01 mol), paraformaldehyde (0.61 g, 0.02 mol), and 35 mL xylene were placed into a 100 mL round-bottom flask equipped with a reflux condenser. The mixture was stirred at 120 °C for 6 hr. When the reaction was complete, the mixture was cooled to room temperature to afford the crude products. Afterward, the purified crystal samples were obtained by recrystallizing from toluene/acetone mixtures (1:1) (yield ca. 90%). ^1^H NMR (CDCl_3_), ppm: *δ* = 1.53–2.04 (m, 8H, -CH_2_-), 3.01–3.14 (m, 2H), 4.66 (d, 2H, Ar-CH_2_-N, oxazine), 5.35–5.36 (d, 2H, O-CH_2_-N, oxazine), 6.88–7.33 (8H, Ar). IR spectra (KBr), cm^−1^: 1773, 1708 (imide I), 1495 (stretching of trisubstituted benzene ring), 1386 (imide II), 1228 (C-O-C asymmetric stretching), 925 (oxazine ring related mode). Anal. calcd. for C_22_H_22_N_2_O_3_: C, 72.91; H, 6.12; N, 7.73. Found: C, 72.85%; H, 6.15%; N, 7.70%.

### 2.8. Synthesis of 2-(3-Phenyl-3,4-dihydro-2H-benzo[e][1,3]oxazin-8-yl)-isoindoline-1,3-dione (oPP-a)

The monomer was synthesized by treating 2-(2-hydroxyphenyl)-isoindoline-1,3-dione (*o*-PP) with aniline and formaldehyde in a 1:1:2 molar ratio to generate the *ortho*-substituted benzoxazine (*o*PP-a). The crude products were prepared following the previously reported method [21], and the purified crystal samples were obtained by recrystallizing them from the toluene/acetone mixtures (1:1). (yield ca. 91%). ^1^H NMR (DMSO), ppm: *δ* = 4.72 (s, Ar-CH_2_-N, oxazine), 5.42 (s, O-CH_2_-N, oxazine), 6.85–7.96 (12H, Ar). IR spectra (KBr), cm^−1^: 1772, 1720 (imide I), 1497 (stretching of trisubstituted benzene ring), 1385 (imide II), 1231 (C-O-C asymmetric stretching), 924 (oxazine ring-related mode). Anal. calcd. for C_22_H_16_N_2_O_3_: C, 74.15; H, 4.53; N, 7.86. Found: C, 74.12%; H, 4.58%; N, 7.81%.

### 2.9. Polymerization of Imide Functional Benzoxazines 

The benzoxazine monomers were thermally polymerized at different temperature cycles without any initiators or catalysts, in an air-circulating oven. The samples were polymerized stepwise at 180, 200, 220, and 240 °C for 1 h each, and then slowly cooled to room temperature. Dark brown polybenzoxazine films were obtained. 

## 3. Result and Discussion

### 3.1. Synthesis and Atropisomerism of ortho-Imide Functional Benzoxazines 

**Scheme 2:**
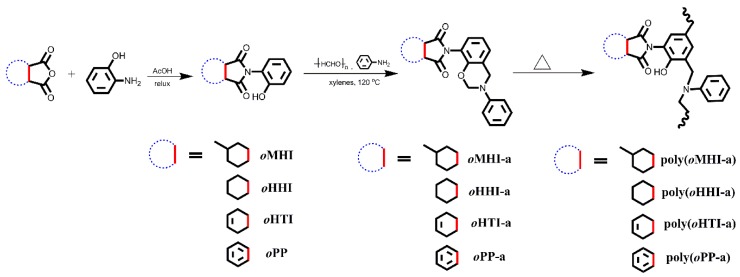
Synthesis and polymerization of *ortho*-imide functional benzoxazine monomers.

A class of *ortho*-imide benzoxazine monomers with different end-caps (Scheme 2) were synthesized by using the *ortho*-imide phenols paraformaldehyde and aniline. Various end-caps with different electronic characteristics and rigidities for 4-methyl-hexahydrophthalimide, hexahydrophthalimide, tetrahydrophthalimide, and phthalimide in the *ortho*-position regarding the oxygen of the oxazine ring were designed in benzoxazine monomers. These compounds were highly purified prior to their use, in order to eliminate impurity-related uncertainties. As detailed later, this allowed for the observation of sharp and intense melting endotherms in DSC analysis. 

**Figure 1:**
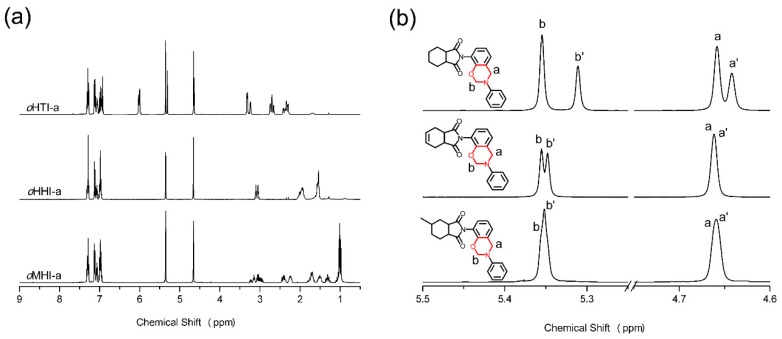
(**a**) ^1^H NMR spectra of *o*MHI-a, *o*HHI-a and *o*HTI-a. (**b**) The expanded region between 4.6 and 5.5 ppm shows the two sets of doublet resonances for these monomers.

**Figure 2:**
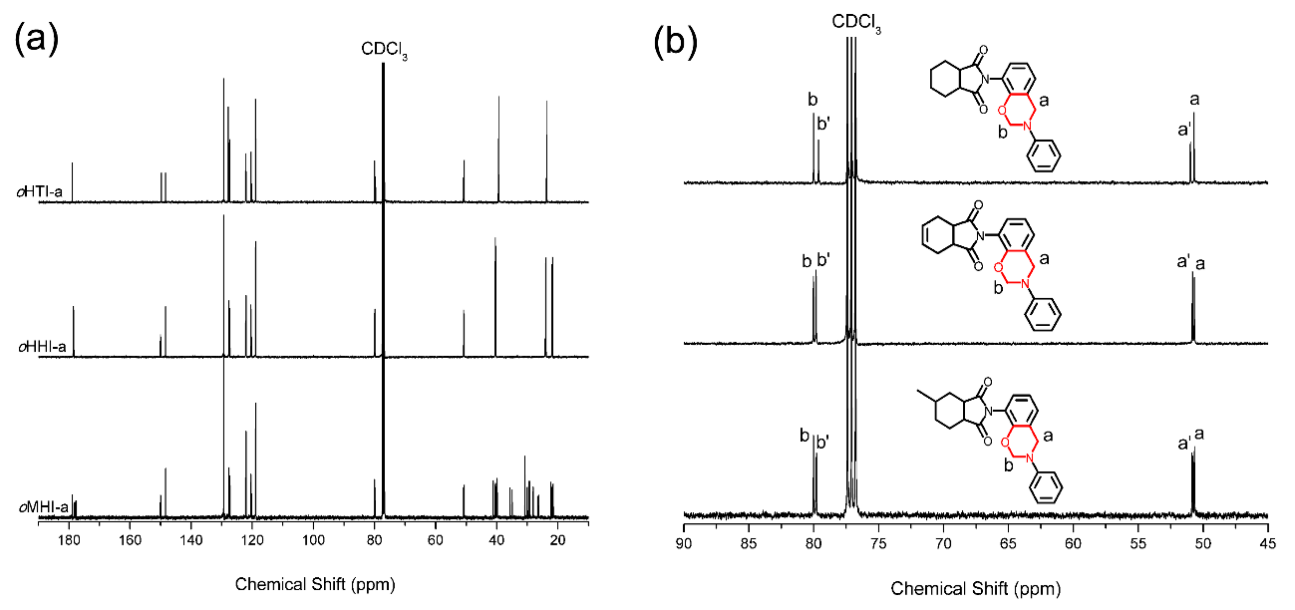
(**a**) ^13^C NMR spectra of *o*-MHI-a, *o*HHI-a and *o*HTI-a. (**b**) The expanded region between 45 and 90 ppm showing the two sets of doublet resonances for these monomers.

The chemical structures of the monomers were confirmed by using ^1^H and ^13^C NMR and FTIR. Besides the two-dimensional (2D) NMR technique, heteronuclear multiple quantum coherence spectroscopy (HMQC) was employed to study the local proton–carbon proximity, and identify the atropisomers. As shown in Figure 1, the characteristic proton resonance signals due to the O-C*H*_2_-N and the Ar-C*H*_2_-N groups in the oxazine ring of *o*MHI-a, *o*HHI-a and *o*HTI-a were observed as two sets of doublet resonances in a range of 5.31–5.36 ppm and 4.64–4.66 ppm, respectively. In addition, ^13^C NMR analyses were also performed, to further confirm the structures of the benzoxazine monomers. As seen in Figure 2, two set of doublet resonances at around 50 and 80 ppm were observed, which are typical carbon resonances of Ar-*C*H_2_-N- and -O-*C*H_2_-N- of the oxazine ring, respectively. Moreover, the oxazine protons were correlated to four different carbon resonances in the ^1^H-^13^C HMQC spectra (Figures S1–S3 of Supporting Information), suggesting the co-existence of two isomers in *o*MHI-a, *o*HHI-a, and *o*HTI-a.

**Figure 3:**
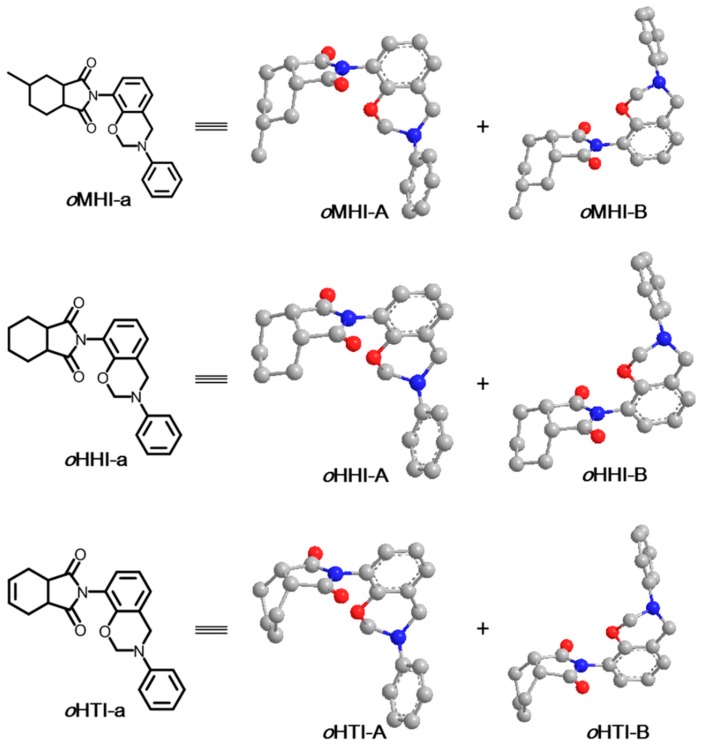
Chemical structures of *o*MHI-a, *o*HHI-a, and *o*HTI-a. The structures of these three benzoxazine monomers were obtained by DFT calculations.

To interpret the NMR spectra, DFT calculations were performed on benzoxazine monomers using the B3LYP/6-31+G(d) method, which identified two configurations for *o*MHI-a, *o*HHI-a, and *o*HTI-a, respectively. As shown in Figure 3, the oxazine six-membered ring in each monomer was perpendicular to the plane of the imide five-membered ring, due to the repulsion between the oxygen atoms in both the oxazine and imide rings. Besides, the alicyclic end-caps of *o*MHI-a, *o*HHI-a, and *o*HTI-a can be located on the either side of oxazine ring, relative to the imide five-membered ring, resulting in two configurations for each monomer. In all of these structures, the alicyclic end-caps attached to imide have characteristic intramolecular repulsions between the oxygen atoms, which hinders their rotation about the C–N bond in a way that is similar to that in *ortho*-tetrahydrophthalimide benzoxazines [27]. 

**Figure 4:**
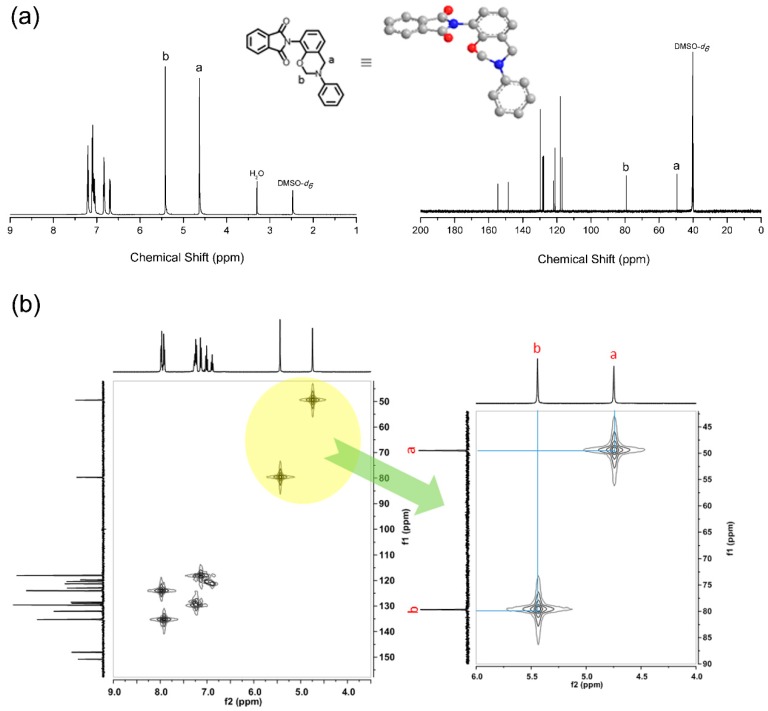
(**a**) ^1^H and ^13^C NMR spectra of *o*PP-a. The inset is the DFT-optimized structure of *o*PP-a. (**b**) HMQC NMR spectra of *o*PP-a in DMSO-*d_6_*. The expanded view shows that the oxazine protons of *o*PP-a are correlated to two different carbon resonances.

In a different set of experiments, we synthesized an *ortho*-phthalimide functional benzoxazine, *o*PP-a, by replacing the alicyclic end-caps in *o*MHI-a, *o*HHI-a, and *o*HTI-a with an aromatic benzene ring, which showed dramatically different NMR spectra when compared with the benzoxazines with alicyclic end-caps. As seen in Figure 4a, two singlet resonances were observed at 5.42 and 4.72 ppm, which can be attributed to O-C*H*_2_-N and the Ar-C*H*_2_-N of the oxazine rings, respectively. Besides, a set of singlet resonances at 50 and 79 ppm were also present, which are typical carbon resonances of Ar-*C*H_2_-N- and -O-*C*H_2_-N- of the oxazine ring, respectively. Additionally, as shown in Figure 4b, each oxazine proton of -C*H*_2_- is correlated to the corresponding singlet carbon resonance in the ^1^H–^13^C HMQC spectra. All of these features suggested the absence of isomerism in *o*PP-a, thus ruling out the atropisomerism of benzoxazine when a benzene ring is attached to the imide. To confirm this, we also performed DFT calculations, which showed only one optimized structure (see Figure 4a). In this molecule, although the intramolecular repulsion still exists between the oxygen atoms in the imide and the oxazine ring, which hinders the rotation about the C–N bond, the atropisomerism disappears because the end-capped aromatic six-membered ring is in the same plane as the imide five-membered ring. In other words, the rotation of the end-cap above the C-N bond by 180° gives rise to the same structure. Moreover, the NMR measurements, along with the DFT calculations, provide further support for the absence of intramolecular hydrogen bonding formed of -C=O…HAr in *o*PP-a [29]. 

Our experiments showed that atropisomerism in *ortho*-imide benzoxazines could be fully controlled by the steric configurations of the end-caps. This unique atropisomerism in *ortho*-imide functional benzoxazines occurred because the end-caps were in a different plane of the imide five-membered ring, allowing for atropisomerization to be switched on/off by the manipulation of the end-caps. The combined experimental and computational analyses in this work therefore provide further insights into the atropisomerization mechanism at the molecular level. 

### 3.2. Polymerization Behaviors of Benzoxazine Monomers

**Figure 5:**
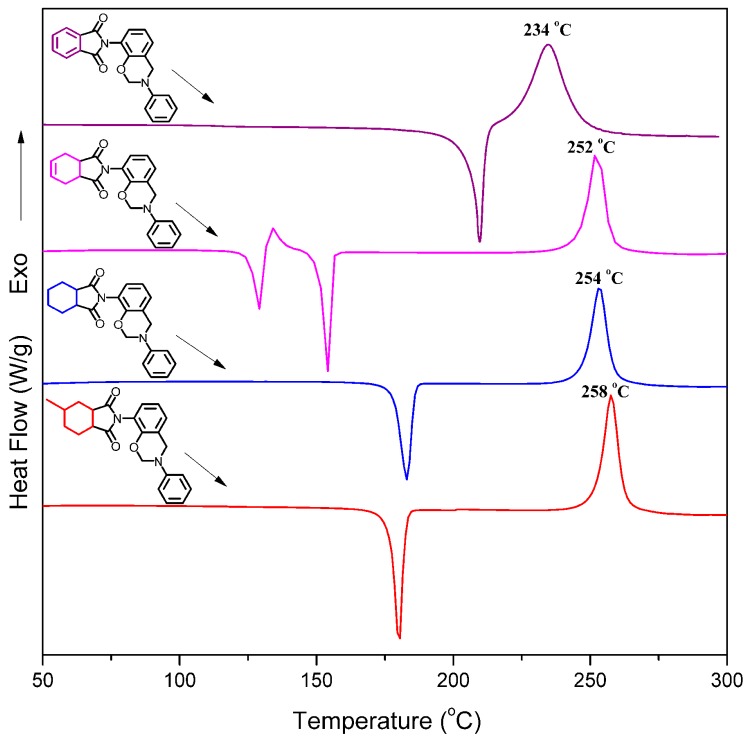
DSC thermograms of *ortho*-imide functional benzoxazine monomers.

**Figure 6:**
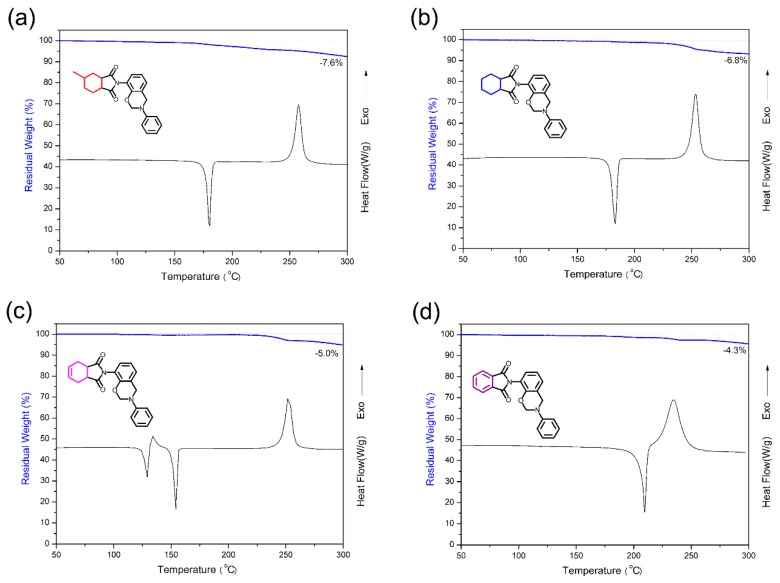
TGA vs DSC plots of benzoxazine monomers (heating rate: 10 °C/min, under a N_2_ atmosphere): (**a**) *o*MHI-a, (**b**) *o*HHI-ac, (**c**) *o*HTI-a, and (**d**) *o*PP-a.

The end-caps attached to the imide may also influence the thermally activated polymerization of the monomers and offer flexibility in the design of new thermosets. The polymerization behaviors of benzoxazines are depicted in Figure 6, and the results are summarized in Table 1. Since impurities and byproducts can act as an initiators and/or catalysts for the ring-opening polymerization of the oxazine ring [30], it is important to use high-purity monomers in order to obtain mechanistic insights into the polymerization process [31]. Purification by recrystallization was found to be highly successful in this work, as indicated from the very sharp endothermic peaks in the DSC measurements (see Figure 5).

**Scheme 3:**
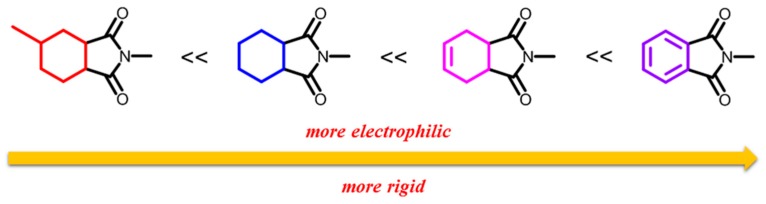
Electrophilicity and rigidity orders of the imide end-caps.

It is generally expected that the melting temperature rises as the rigidity of monomers increases. According to the rigidity order, 4-methyl-hexahydrophthalimide < hexahydrophthalimide < tetrahydrophthalimide < phthalimide (Scheme 3), the rigidities of the benzoxazine monomers should follow the order of *o*MHI-a < *o*HHI-a < *o*HTI-a < *o*PP-a, and so did the melting points. However, the DSC measurements showed a different order for the melting points: *o*HTI-a < *o*MHI-a < *o*HHI-a < *o*PP-a (see Figure 5). Among these monomers, *o*HTI-a was the only one that has a crystal-to-crystal phase-transition [27,32]; hence, the unexpected low melting temperature of *o*HTI-a can be attributed to the formation of less stable crystalline structures. Besides, the thermograms showed that the maxima of the ring-opening polymerization for *o*MHI-a, *o*HHI-a, *o*HTI-a, and *o*PP-a centers at 258, 254, 252, and 234 °C, respectively, which were all lower than that of PH-a (a phenol- and aniline-based benzoxazine monomer) [33]. This suggests that the incorporation of the imide group into benzoxazine reduces the ring-opening polymerization temperature of the oxazine ring, e.g., through electro-withdrawal at the *ortho*-position of the oxazine ring. On the other hand, the order of the polymerization temperature for these benzoxazines showed an opposite trend to electronegativity (4-methyl-hexahydrophthalimide < hexahydrophthalimide < tetrahydrophthalimide < phthalimide), which could be interpreted by a mechanism that is proposed by Andreu et al. [34]. The electron-withdrawing groups substituted in the phenolic components could lead to a more acidic phenol species, and therefore, a stronger catalytic effect takes place in the ring-opening polymerization of the benzoxazines. In addition, the heat of polymerization for the *ortho*-imide functional benzoxazine monomers were in the range of 201–226 J/g, suggesting similar thermal events in the ring-opening reaction of the *ortho*-imide benzoxazines.

A comparison between the TGA and DSC thermograms of the benzoxazine monomers is shown in Figure 6. Evaporation may take place prior to polymerization, but we observed no mass loss for *o*HHI-ac, *o*HTI-a, and *o*PP-a before reaching the onset temperatures of polymerization, and there was a marginal weight loss for *o*MHI-a (~4.5%). This is due to the high molecular weight and strong intermolecular interactions between the monomers, which indicates the high melting temperatures and thermal stability during the thermal treatment. Above the onset temperature of polymerization, there was a further weight loss of 7.6% for *o*MHI-a. Weight losses were also observed for *o*HHI-ac (6.8%), *o*HTI-a (5.0%), and *o*PP-a (4.3%), which were due to the cleavage of the zwitterionic intermediate, and the formation of very unstable phenolic species and *N*-methyleneaniline [11]. However, the weight losses during the polymerization of these monomers were significantly lower than those of other reported monofunctional benzoxazines [11,35]. 

**Figure 7:**
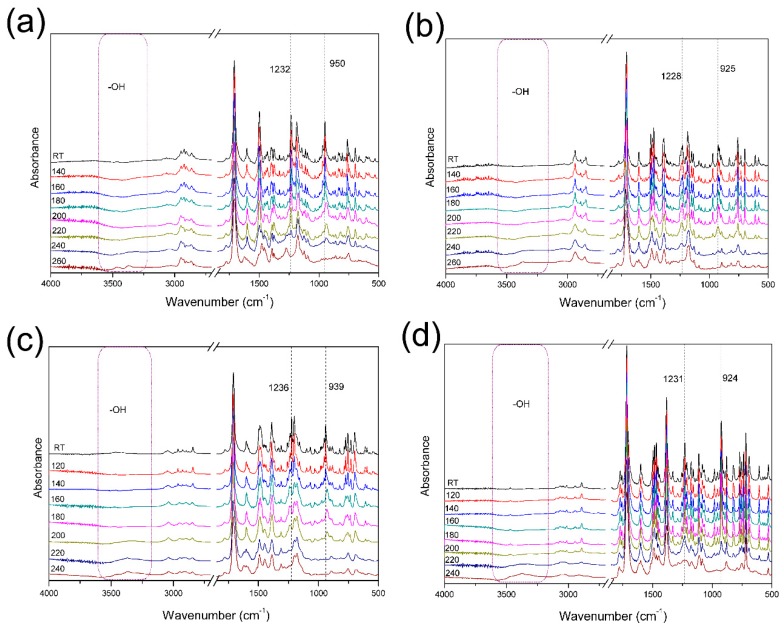
FTIR spectra of the benzoxazine monomers after various thermal treatments: (**a**) *o*MHI-a, (**b**) *o*HHI-ac, (**c**) *o*HTI-a, and (**d**) *o*PP-a.

FTIR analyses were also carried out, in order to qualitatively study the structural evolution. The spectra of the benzoxazine monomers are shown in Figure S4, and their spectra after various thermal treatments are presented in Figure 7. The characteristic absorption bands at 1232 and 1228 cm^−1^ (C-O-C asymmetric stretching modes) [36], and 950 and 925 cm^−1^ (benzoxazine-related mode) gradually disappeared in the temperature region from room temperature to 260 °C, for both *o*MHI-a and *o*HHI-a [37]. Meanwhile, the broad-band -OH stretching mode emerged at around 3400 cm^−1^, which was the fingerprint for the ring-opening reactions of benzoxazine. Besides, the benzoxazine characteristic bands for *o*HTI-a and *o*PP-a were found to disappear at a lower temperature (240 °C), which is consistent with the DSC measurements.

### 3.3. Thermal Properties of the Ortho-Imide Functional Polybenzoxazines

**Figure 8:**
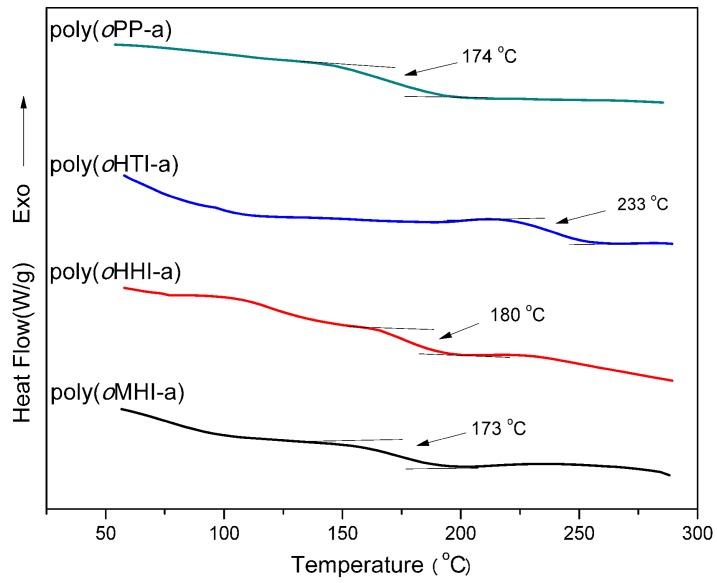
DSC thermograms of polybenzoxazines recorded under nitrogen at a heating rate of 10 °C/min.

Figure 8 shows the DSC thermograms of the heat-treated polybenzoxazines, with the heating schedule stated in the experimental section. For the polybenzoxazines derived from the *ortho*-imide functional benzoxazine monomers with different end-caps, the glass transition temperature (*T_g_*) showed an order of poly(*o*MHI-a) < poly(*o*HHI-ac) < poly(*o*HTI-a), which was consistent with the rigidity order of 4-methyl-hexahydrophthalimide < hexahydrophthalimide < tetrahydrophthalimide. Poly(*o*PP-a) had a much lower *T_g_* temperature (174 °C) than that of poly(*o*HTI-a), although poly(*o*PP-a) bore a much more rigid aromatic end-cap, suggesting a trade-off effect between the rigidity of end-caps and *T_g_* temperatures of polybenzoxazines. As the rigidities of the end-caps in imide functionality increased, the crosslinking density of polybenzoxazine declined, due to the steric hindrance, leading to a low *T_g_* temperature. As a result, poly(*o*HTI-a) exhibited the highest *T_g_* temperature among this class of polybenzoxazines. 

**Figure 9:**
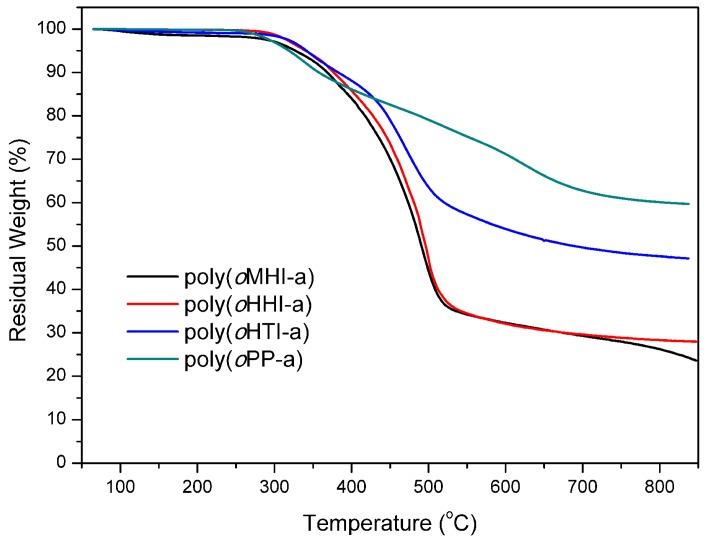
TGA thermograms of poly(*o*MHI-a), poly(*o*HHI-ac), poly(*o*HTI-a), and poly(*o*PP-a).

**Figure 10:**
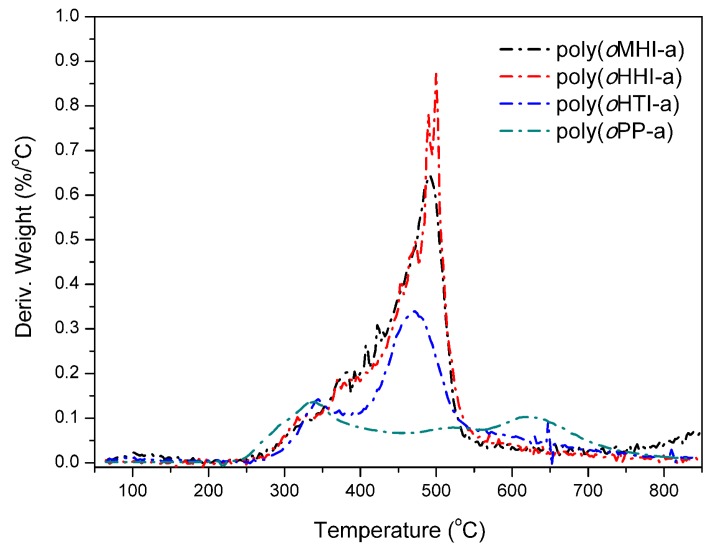
Derivative weigh loss of poly(*o*MHI-a), poly(*o*HHI-ac), poly(*o*HTI-a), and poly(*o*PP-a).

The thermal stability of polybenzoxazines were studied by thermogravimetric analysis (TGA) under nitrogen atmosphere. The weight loss and the derivate weight loss curves are shown in Figure 9 and Figure 10, and values of 5% and 10% weight loss temperatures (T_5_ and T_10_) and the char yield (Yc) at 800 °C are summarized in Table 2. Poly(*o*HTI-a) shows the highest T_5_ and T_10_ values, but poly(*o*PP-a) exhibits the highest Yc value. According to the derivate weight loss, poly(*o*MHI-a), poly(*o*HHI-a), and poly(*o*HTI-a) showed a high-temperature weight-loss stage, in the range of 450–500 °C. Poly(*o*PP-a) showed a stable weight loss rate in this region, but a high-temperature weight-loss stage, from 300 to 400 °C. This unique degradation range can be attributed to the thermal conversion of *ortho*-hydroxypolyimides into polybenzoxazoles [21,38], which results in a much higher Yc value compared with other polybenzoxazines containing cyclohexane or cyclohexene end-caps. The TGA results also indicate that the thermal conversion of benzoxazole formation can only take place in *ortho*-hydroxyimide, with aromatic end-caps. Moreover, the broadening of the derivative peaks in Figure 10 suggested a slow decomposition rate over a wide temperature range, which, in general, is beneficial from the flammability point of view [39,40].

Finally, we looked at the flame-retardant capability of the polymers, by the limiting oxygen index (LOI) tests, which were the minimum percentages of oxygen in a mixture of oxygen and nitrogen that was present in the continuous combustion of the polymers. The LOI was typically used to quantify the flame-retardant capabilities of the polymeric materials. This parameter can be determined from the Yc values, based on TGA results by using the Van Krevelen equation [41]:

LOI (%) = 17.5 + 0.4Yc(1)

Herein, the LOI values of poly(*o*MHI-a), poly(*o*HHI-a), poly(*o*HTI-a), and poly(*o*PP-a) at 800 °C were found to 27.9, 28.7, 36.7, and 41.5, respectively. Poly(*o*MHI-a) has a LOI value that is slightly lower than 28, which falls in the slow burning region (21 < LOI < 28); but poly(*o*HHI-a), poly(*o*HTI-a), and poly(*o*PP-a) all have LOI values in the self-extinguishing region (LOI > 28) [42], suggesting that the polybenzoxazines derived from *ortho*-imide functional benzoxazines were good candidates for applications such as fire-resistant and electronic encapsulation materials. 

## 4. Conclusions

A series of *ortho*-imide functional benzoxazine monomers with different end-caps were successfully synthesized, which demonstrates the flexibility of manipulating atropisomerization in benzoxazine, i.e., by the steric configuration of the end-caps. Besides, the DSC results show that the end-caps can strongly influence the polymerization temperature of the benzoxazine monomers. *ortho*-Phthalimide benzoxazine, *o*PP-a, exhibits the lowest polymerization among these benzoxazines. Moreover, a trade-off effect has also been found, between the thermal properties and the rigidities of end-caps in the polybenzoxazines. Poly(*o*HTI-a) shows the highest *T_g_* temperatures (233 °C), the highest T_5_ (342 °C) and T_10_ (382 °C) values among these polybenzoxazines, indicating its great potential in applications such as fire-resistant materials, electronic encapsulation materials, and polymeric matrixes for high-performance composites.

## Figures and Tables

**Table 1:** DSC thermograms of benzoxazine monomers.

Monomer	Melting Temp (°C)	Max Temp (°C)	Heat of Polymerization (J/g)
*o*MHI-a	179	258	201
*o*HHI-a	183	254	218
*o*HTI-a	154	252	226
*o*PP-a	209	234	214

**Table 2:** Thermal properties of *ortho*-imide functional polybenzoxazines.

Polybenzoxazine Based on	*T_g_*/DSC (°C)	T_5_ (°C)	T_10_ (°C)	Yc (wt %)	LOI (%)
*o*MHI-a	173	327	367	26	27.9
*o*HHI-a	180	340	375	28	28.7
*o*HTI-a	233	342	382	48	36.7
*o*PP-a	174	319	358	60	41.5

## Supplementary Materials

The following are available online at https://www.mdpi.com/2073-4360/11/3/399/s1, Figure S1. ^1^H-^13^C HMQC 2D NMR spectrum for *o*MHI-a, Figure S2. 1H-13C HMQC 2D NMR spectrum of *o*HHI-a, Figure S3. 1H-13C HMQC 2D NMR spectrum of *o*HTI-a, Figure S4. FTIR spectra of benzoxazine monomers.

## References

[1] De Abajo J., De la Campa J.G., Kricheldorf H.R. (1999). Processable aromatic polyimides. Progress in Polyimide Chemistry.

[2] Vanherck K., Koeckelberghs G., Vankelecom I.F.J. (2013). Crosslinking polyimides for membrane applications: A review. Prog. Polym. Sci..

[3] Wang M.W., Lin C.H., Juang T.Y. (2013). Steric hindrance control synthesis of primary amine-containing benzoxazines and properties of the resulting poly(benzoxazine imide) thermosetting films. Macromolecules.

[4] And G.C.E., Paprotny J., Irwin R.S. (1996). Melt-processable poly(ether imide)s based on catechol bis(ether anhydride). Macromolecules.

[5] Hu Z., Wang M., Li S., Liu X., Wu J. (2005). Ortho alkyl substituents effect on solubility and thermal properties of fluorenyl cardo polyimides. Polymer.

[6] Holly F.W., Cope A.C. (1944). Condensation products of aldehydes and ketones with o-aminobenzyl alcohol and o-hydroxybenzylamine. J. Am. Chem. Soc..

[7] Ishida H., Agag T. (2011). Handbook of Benzoxazine Resins.

[8] Ishida H., Froimowicz P. (2017). Advanced and Emerging Polybenzoxazine Science and Technology.

[9] Ghosh N.N., Kiskan B., Yagci Y. (2007). Polybenzoxazines-new high performance thermosetting resins: Synthesis and properties. Prog. Polym. Sci..

[10] Kim H.J., Brunovska Z., Ishida H. (1999). Synthesis and thermal characterization of polybenzoxazines based on acetylene-functional monomers. Polymer.

[11] Sini N.K., Endo T. (2016). Toward elucidating the role of number of oxazine rings and intermediates in the benzoxazine backbone on their thermal characteristics. Macromolecules.

[12] Zhang K., Liu J., Ishida H. (2014). An ultrahigh performance cross-linked polybenzoxazole via thermal conversion from poly(benzoxazine amic acid) based on smart *o*-benzoxazine chemistry. Macromolecules.

[13] Agag T., Liu J., Graf R., Spiess H.W., Ishida H. (2012). Benzoxazole resin: A novel class of thermoset polymer via smart benzoxazine resin. Macromolecules.

[14] Zhang K., Han L., Froimowicz P., Ishida H. (2017). A smart latent catalyst containing o-trifluoroacetamide functional benzoxazine: Precursor for low temperature formation of very high Performance polybenzoxazole with low dielectric constant and high thermal stability. Macromolecules.

[15] Velez-Herrera P., Doyama K., Abe H., Ishida H. (2008). Synthesis and characterization of fluorinated polybenzoxazine material with low dielectric constant. Macromolecules.

[16] Wu J., Xi Y., McCandless G.T., Xie Y., Menon R., Patel Y., Yang D.J., Lacono S.T., Novak B.M. (2015). Synthesis and characterization of partially fluorinated polybenzoxazine resins utilizing octafluorocyclopentene as a versatile building block. Macromolecules.

[17] Wang C.F., Su Y.C., Kuo S.W., Huang C.F., Sheen Y.C., Chang F.C. (2006). Low-surface-free-energy materials based on polybenzoxazines. Angew. Chem. Int. Ed..

[18] Kuo S.W., Wu Y.C., Wang C.F., Jeong K.U. (2009). Preparing low-surface-energy polymer materials by minimizing intermolecular hydrogen-bonding interactions. J. Phys. Chem. C.

[19] Liao C.S., Wang C.F., Lin H.C., Chou H.Y., Chang F.C. (2008). Tuning the surface free energy of polybenzoxazine thin films. J. Phys. Chem. C.

[20] Takeichi T., Guo Y., Rimdusit S. (2005). Performance improvement of polybenzoxazine by alloying with polyimide: Effect of preparation method on the properties. Polymer.

[21] Zhang K., Liu J., Ohashi S., Liu X., Han Z., Ishida H. (2015). Synthesis of high thermal stability polybenzoxazoles via ortho-imide-functional benzoxazine monomers. J. Polym. Sci. Part A Polym. Chem..

[22] Agag T., Arza C.R., Maurer F.H.J., Ishida H. (2010). Primary Amine-Functional Benzoxazine Monomers and Their Use for Amide-Containing Monomeric Benzoxazines. Macromolecules.

[23] Zhang K., Ishida H. (2015). An Anomalous Trade-off Effect on the properties of smart ortho-functional benzoxazines. Polym. Chem..

[24] Kolanadiyil S.N., Bijwe J., Varma I.K. (2013). Synthesis of itaconimide/nadimide-functionalized benzoxazine monomers: Structural and thermal characterization. React. Funct. Polym..

[25] Zhang K., Ishida H. (2015). Thermally stable polybenzoxazines via ortho-norbornene functional benzoxazine monomers: Unique advantages in monomer synthesis, processing and polymer properties. Polymer.

[26] Zhang K., Yu X. (2018). Catalyst-free and low-temperature terpolymerization in a single-component benzoxazine resin containing both norbornene and acetylene functionalities. Macromolecules.

[27] Zhang K., Shang Z., Evans C.J., Han L., Ishida H., Yang S. (2018). Benzoxazine atropisomers: Intrinsic atropisomerization mechanism and conversion to high performance thermosets. Macromolecules.

[28] Frisch M.J., Trucks G.W., Schlegel H.B., Scuseria G.E., Robb M.A., Cheeseman J.R., Montgomery J.A., Vreven T., Kudin K.N., Burant J.C. (2004). Gaussian 03, Revision E.01.

[29] Froimowicz P., Zhang K., Ishida H. (2016). Intramolecular hydrogen bonding in benzoxazines: When structural design becomes functional. Chem. Eur. J..

[30] Zhang K., Froimowicz P., Han L., Ishida H. (2016). Hydrogen-bonding characteristic and unique ring-opening polymerization behavior of *ortho*-methylol functional benzoxazine. J. Polym. Sci. Part A Polym. Chem..

[31] Ohashi S., Iguchi D., Heyl T.R., Froimowicz P., Ishida H. (2018). Quantitative studies on the *p*-substituent effect of the phenolic component on the polymerization of benzoxazines. Polym. Chem..

[32] Burger A., Ramberger R. (1979). On the polymerization of pharmaceuticals and other molecular ctrystals. Microchim. Acta.

[33] Han L., Zhang K., Ishida H., Froimowicz P. (2017). Study of the effects of intramolecular and intermolecular hydrogen-bonding systems on the polymerization of amide-containing benzoxazines. Macromol. Chem. Phys..

[34] Andreu R., Reina J.A., Ronda J.C. (2008). Studies on the thermal polymerization of substituted benzoxazine monomers: Electronic effects. J. Polym. Sci. Part A Polym. Chem..

[35] Zhang K., Han L., Nie Y., Szigeti M.L., Ishida H. (2018). Examining the effect of hydroxyl groups on the thermal properties of polybenzoxazines: Using molecular design and Monte Carlo simulation. RSC Adv..

[36] Dunkers J., Ishida H. (1995). Vibrational assignments of 3-alkyl-3, 4-dihydro-6-methyl-2H-1,3-benzoxazines in the Fingerprint Region. Spectrochim. Acta.

[37] Han L., Iguchi D., Gil P., Heyl T.R., Sedwick V.M., Arza C.R., Ohashi S., Lacks D.J., Ishida H. (2017). Oxazine ring-related vibrational modes of benzoxazine monomers using fully aromatically substituted, deuterated, ^15^N isotope exchanged, and oxazine-ring-substituted compounds and theoretical calculations. J. Phys. Chem. A.

[38] El-Mahdy A.F.M., Kuo S.W. (2018). Direct synthesis of poly(benzoxazine imide) from an ortho-benzoxazine: Its thermal conversion to highly cross-linked polybenzxoazole and blending with poly(4-vinylphenol). Polym. Chem..

[39] Wang J., Wu M., Liu W., Yang S., Bai S., Ding Q., Li Y. (2010). Synthesis, curing behavior and thermal properties of fluorine containing benzoxazines. Eur. Polym. J..

[40] Min C., Liu D., Shen C., Zhang Q., Song H., Li S., Shen X., Zhu M., Zhang K. (2018). Unique synergistic effects of graphene oxide and carbon nanotube hybrids on the tribological properties of polyimide nanocomposites. Tribol. Int..

[41] Van Krevelen D.W. (1975). Some basic aspects of flame resistance of polymeric materials. Polymer.

[42] Mallakpour S., Behranvand V. (2015). The influence of acid-treated multi-walled carbon nanotubes on the surface morphology and thermal properties of alanine-based poly(amide-imide)/MWCNT nanocomposites system. Colloid Polym. Sci..

