# Supplementary material for: Effects of End-Caps on the Atropisomerization, Polymerization, and the Thermal Properties of ortho-Imide Functional Benzoxazines
Source: Polymers (Basel). 2019 Mar 1;11(3):399. doi: 10.3390/polym11030399 (PMC6473311; doi:10.3390/polym11030399)
Supplement: Supplementary file 1 [file polymers-11-00399-s001.pdf]

Supplementary Data

Effects of End-Caps on the Atropisomerization, Polymerization and Thermal Properties of *Ortho*-Imide Functional Benzoxazines

Kan Zhang,[1,*] Yuqi Liu,[1] Zhikun Shang,[1] Corey J. Evans,[2] and Shengfu Yang[2,*]

[1] Research School of Polymeric Materials, School of Materials Science and Engineering, Jiangsu University, Zhenjiang 212013, China

[2] Department of Chemistry, University of Leicester, Leicester LE1 7RH, United Kingdom;

[*] Corresponding authors: zhangkan@ujs.edu.cn (K. Z.); sfy1@le.ac.uk (S. Y.)

2D NMR Spectra

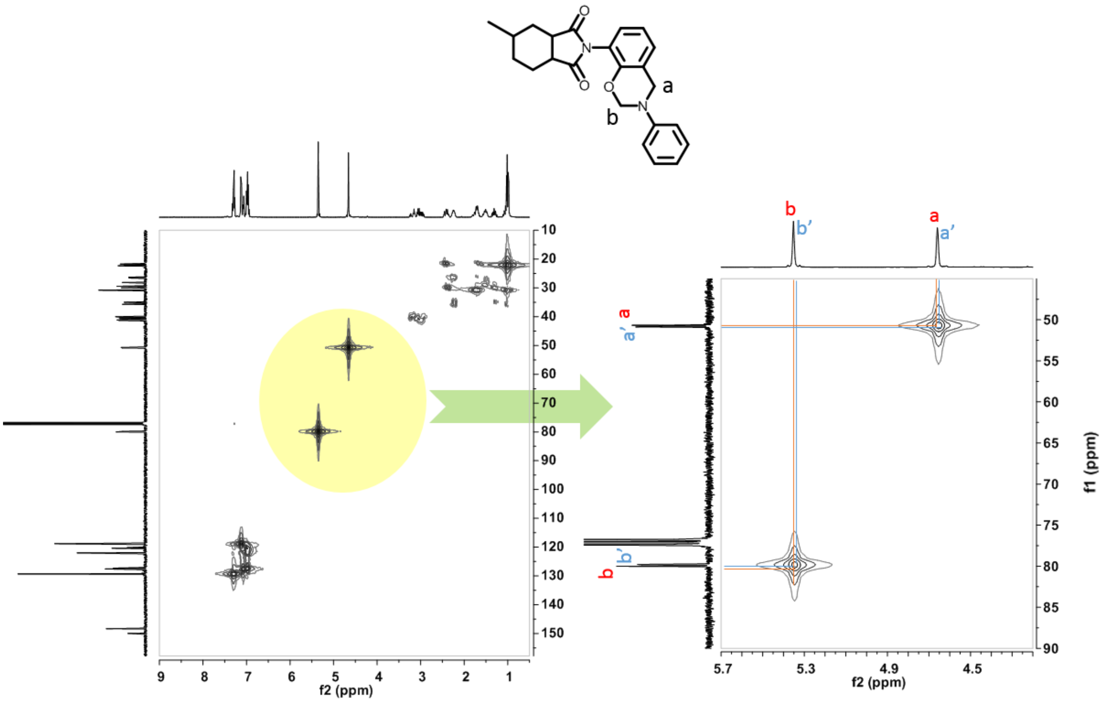

Figure S1. ^{1}H-^{13}C HMQC 2D NMR spectrum of *o*MHI-a.

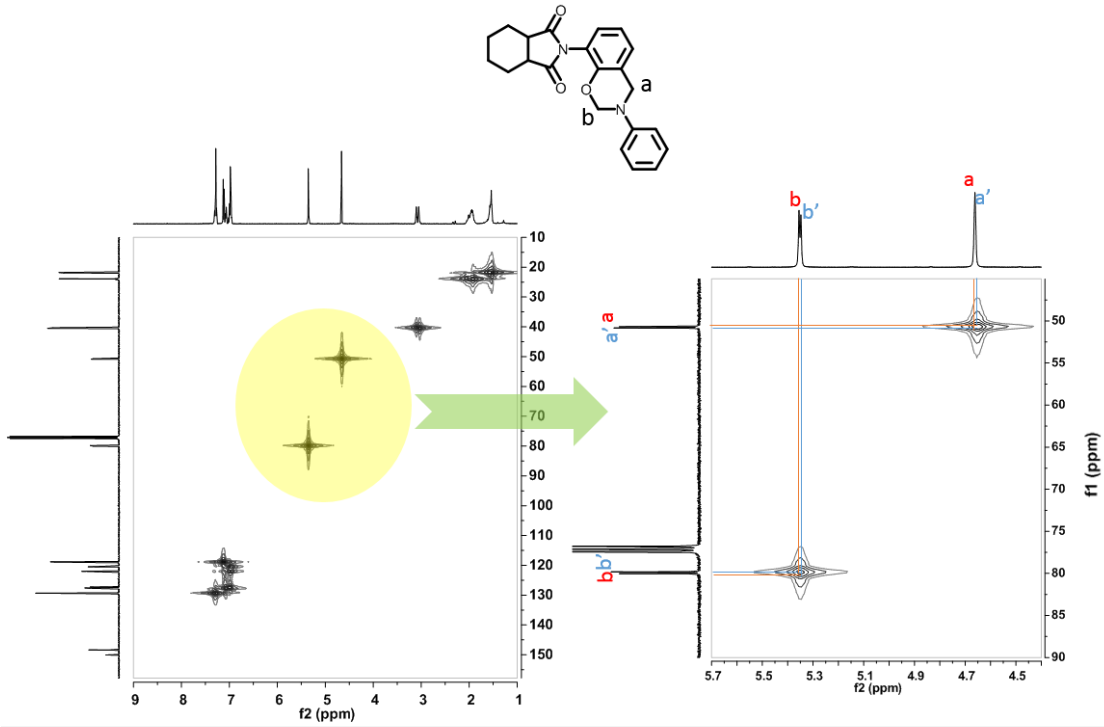

Figure S2. ^{1}H-^{13}C HMQC 2D NMR spectrum of *o*HHI-a.

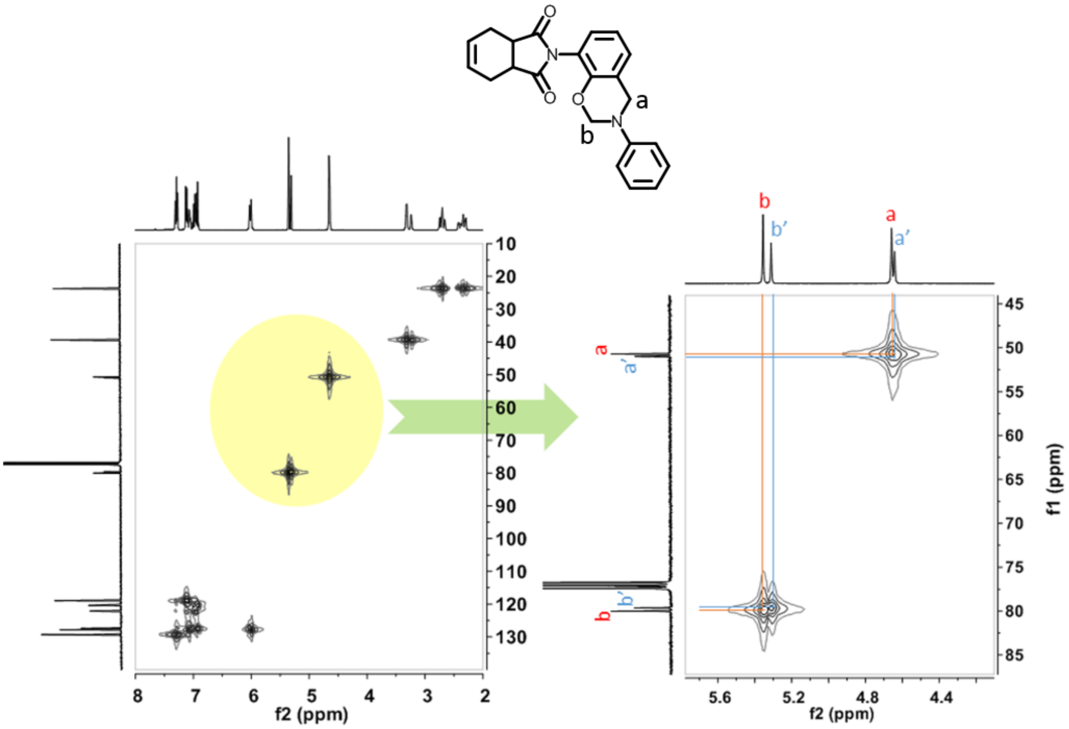

Figure S3. ^{1}H-^{13}C HMQC 2D NMR spectrum of *o*HTI-a.

FT-IR Spectra

In Figure S4, the characteristic doublet at 1779-1772 cm^{-1} and 1720-1708 cm^{-1} are the typical bands for imide, which are attributed to the imide C-C(=O)-C antisymmetric and symmetric stretching, respectively.[1] Besides, the presence of imide is also seen by the other characteristic bands at 1389-1376 cm^{-1}, which is due to the axial stretching of C-N bonding.[1] The bands characteristic of antisymmetric trisubstituted benzene that appear between 1498 and 1488 cm^{-1} confirm the incorporation of imide group into benzoxazine moieties. In addition, the existence of the benzoxazine ring aromatic ether in the monomers is supported by the bands centered in the range of 1236-1228 cm^{-1} due to the C-O-C antisymmetric stretching modes.[2] Furthermore, the characteristic bands at 950, 925, 924 and 939 cm^{-1} for the *o*MHI-a, *o*HHI-a, *o*HTI-a and *o*PP-a, respectively, which are mainly related to the oxazine ring, the vibration of $O-C_2$ vibration with a minor contribution from the phenolic ring (C-H out of plane bending, C-C-C-C torsion).[3]

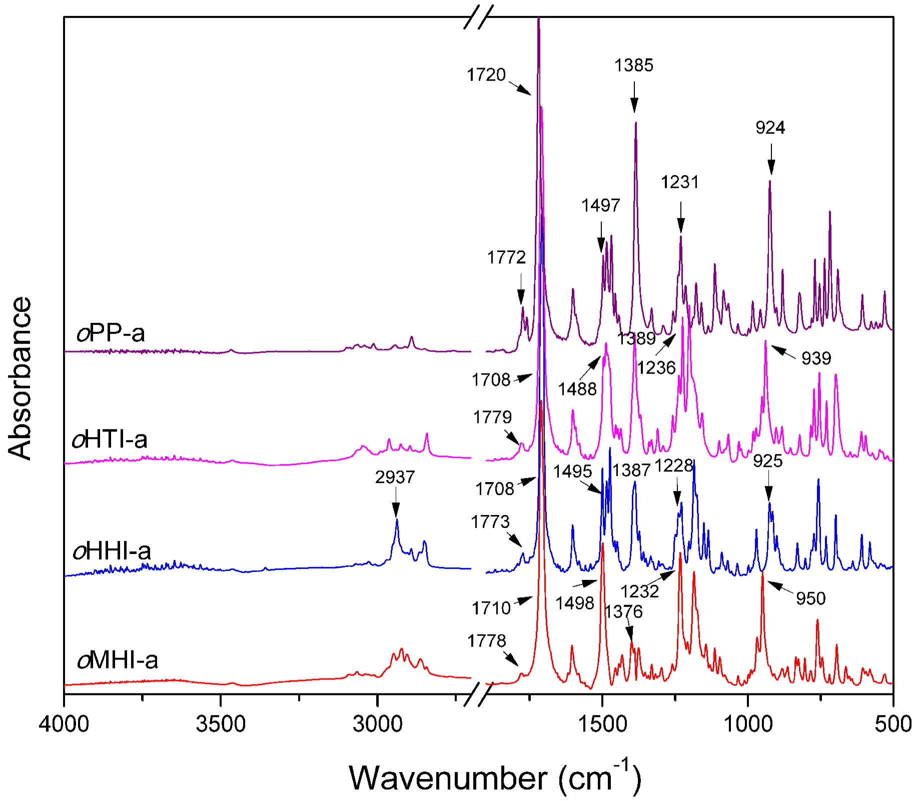

Figure S4. FTIR spectra of benzoxazine monomers.

Atomic Coordinates of the Atoms in Calculated Equilibrium Structures

***o*HMI-a-A**

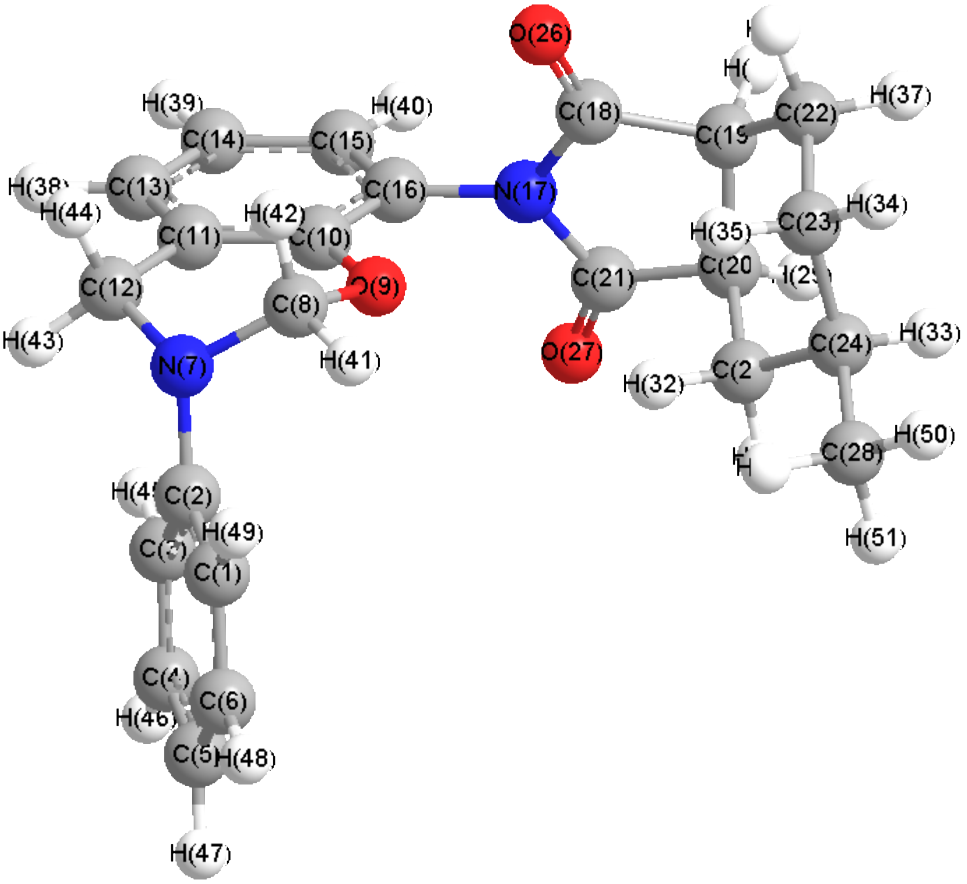

**C(1)**	**3.5264**	**-2.3442**	**-0.8760**
**C(2)**	**3.3715**	**-0.9989**	**-0.4987**
**C(3)**	**3.7638**	**-0.6113**	**0.7913**
**C(4)**	**4.3036**	**-1.5492**	**1.6771**
**C(5)**	**4.4425**	**-2.8862**	**1.3016**
**C(6)**	**4.0450**	**-3.2788**	**0.0189**
**N(7)**	**2.8469**	**-0.0827**	**-1.4630**
**C(8)**	**1.4891**	**-0.2832**	**-1.8762**
**O(9)**	**0.5208**	**0.1722**	**-0.8934**
**C(10)**	**0.7648**	**1.4158**	**-0.3870**
**C(11)**	**2.0097**	**2.0542**	**-0.5248**
**C(12)**	**3.1117**	**1.3512**	**-1.2985**
**C(13)**	**2.1872**	**3.3173**	**0.0484**
**C(14)**	**1.1600**	**3.9435**	**0.7554**
**C(15)**	**-0.0736**	**3.3022**	**0.8885**
**C(16)**	**-0.2733**	**2.0473**	**0.3191**
**N(17)**	**-1.5308**	**1.3817**	**0.4437**
**C(18)**	**-2.4467**	**1.2489**	**-0.6140**
**C(19)**	**-3.6984**	**0.5808**	**-0.0418**

C(20)	-3.1819	-0.1095	1.2402
C(21)	-1.9436	0.7005	1.6054
C(22)	-4.4942	-0.2858	-1.0280
C(23)	-3.9369	-1.7073	-1.1814
C(24)	-3.7422	-2.3932	0.1817
C(25)	-2.7392	-1.5811	1.0166
O(26)	-2.2734	1.6621	-1.7416
O(27)	-1.3624	0.7302	2.6702
C(28)	-3.2799	-3.8480	0.0312
H(29)	-3.9070	-0.0650	2.0591
H(30)	-4.3356	1.4274	0.2575
H(31)	-2.5708	-2.0443	1.9958
H(32)	-1.7706	-1.5944	0.4955
H(33)	-4.7129	-2.3944	0.7053
H(34)	-4.6186	-2.2967	-1.8090
H(35)	-2.9715	-1.6786	-1.7089
H(36)	-4.5281	0.2262	-1.9966
H(37)	-5.5295	-0.3505	-0.6660
H(38)	3.1519	3.8116	-0.0545
H(39)	1.3172	4.9210	1.2015
H(40)	-0.8885	3.7678	1.4345
H(41)	1.2637	-1.3388	-2.0143
H(42)	1.3027	0.2730	-2.8025
H(43)	4.0827	1.4824	-0.8139
H(44)	3.2023	1.7885	-2.3038
H(45)	3.6284	0.4136	1.1218
H(46)	4.5982	-1.2289	2.6734
H(47)	4.8566	-3.6126	1.9956
H(48)	4.1590	-4.3134	-0.2953
H(49)	3.2613	-2.6462	-1.8861
H(50)	-4.0037	-4.4348	-0.5479
H(51)	-3.1589	-4.3324	1.0081
H(52)	-2.3143	-3.9013	-0.4891

***o*HMI-a-B**

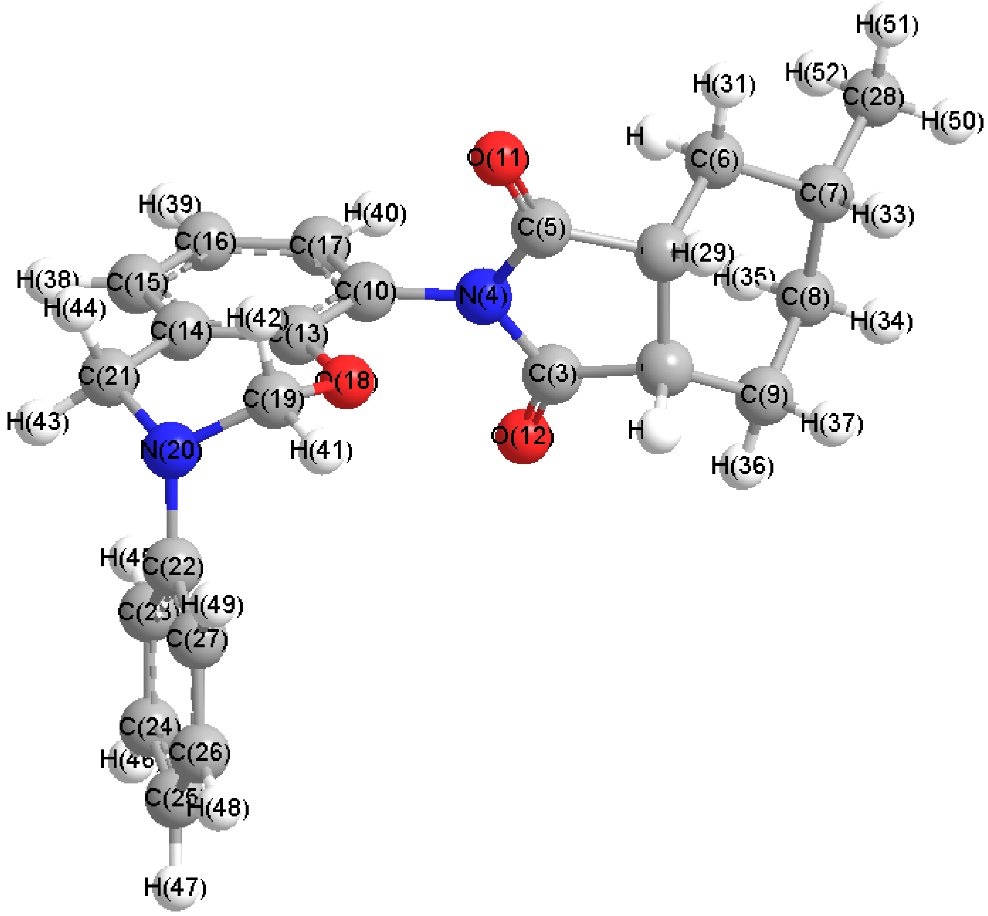

C(1)	-3.1051	-0.5577	-1.1442
C(2)	-2.6468	-1.5257	-0.0308
C(3)	-1.7381	-0.6601	0.8423
N(4)	-1.3244	0.4294	0.0539
C(5)	-2.0000	0.4926	-1.1781
C(6)	-4.4479	0.1564	-0.8345
C(7)	-5.5268	-0.7929	-0.2891
C(8)	-5.0031	-1.4733	0.9872
C(9)	-3.7413	-2.3028	0.7136
C(10)	-0.2736	1.3238	0.4224
O(11)	-1.7696	1.2978	-2.0565
O(12)	-1.3785	-0.8707	1.9816
C(13)	0.9835	1.1816	-0.1895
C(14)	2.0326	2.0533	0.1511
C(15)	1.7981	3.0522	1.1012
C(16)	0.5528	3.1903	1.7157
C(17)	-0.4846	2.3208	1.3712
O(18)	1.1397	0.1637	-1.0840
C(19)	2.3260	0.2626	-1.9163
N(20)	3.5123	0.5664	-1.1707

**C(21)**	**3.3778**	**1.8812**	**-0.5329**
**C(22)**	**4.1033**	**-0.5047**	**-0.4307**
**C(23)**	**4.1905**	**-0.5110**	**0.9696**
**C(24)**	**4.8134**	**-1.5724**	**1.6338**
**C(25)**	**5.3396**	**-2.6495**	**0.9192**
**C(26)**	**5.2474**	**-2.6514**	**-0.4770**
**C(27)**	**4.6470**	**-1.5859**	**-1.1459**
**C(28)**	**-6.8433**	**-0.0452**	**-0.0425**
**H(29)**	**-3.1707**	**-1.0490**	**-2.1202**
**H(30)**	**-1.9530**	**-2.2543**	**-0.4777**
**H(31)**	**-4.7888**	**0.6651**	**-1.7438**
**H(32)**	**-4.2795**	**0.9445**	**-0.0847**
**H(33)**	**-5.7135**	**-1.5767**	**-1.0420**
**H(34)**	**-5.7774**	**-2.1236**	**1.4153**
**H(35)**	**-4.7915**	**-0.7020**	**1.7436**
**H(36)**	**-3.3213**	**-2.6929**	**1.6478**
**H(37)**	**-4.0179**	**-3.1725**	**0.1021**
**H(38)**	**2.6105**	**3.7257**	**1.3691**
**H(39)**	**0.3900**	**3.9662**	**2.4578**
**H(40)**	**-1.4624**	**2.4079**	**1.8349**
**H(41)**	**2.3977**	**-0.7059**	**-2.4073**
**H(42)**	**2.1447**	**1.0537**	**-2.6535**
**H(43)**	**4.2022**	**2.0235**	**0.1708**
**H(44)**	**3.5006**	**2.6432**	**-1.3166**
**H(45)**	**3.7531**	**0.2947**	**1.5505**
**H(46)**	**4.8675**	**-1.5595**	**2.7195**
**H(47)**	**5.8170**	**-3.4750**	**1.4400**
**H(48)**	**5.6641**	**-3.4758**	**-1.0505**
**H(49)**	**4.6183**	**-1.5719**	**-2.2325**
**H(50)**	**-7.6209**	**-0.7256**	**0.3262**
**H(51)**	**-7.2154**	**0.4240**	**-0.9617**
**H(52)**	**-6.7113**	**0.7467**	**0.7068**

*o*HHI-a-A

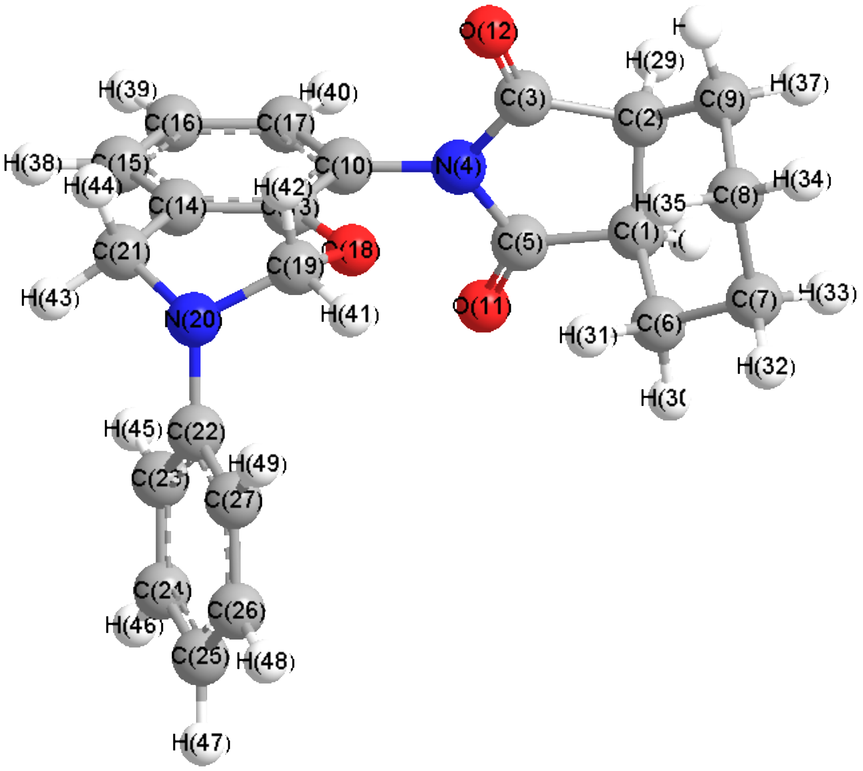

**C(1)**	**3.2649**	**-0.6499**	**-1.2432**
**C(2)**	**3.8520**	**-0.0348**	**0.0482**
**C(3)**	**2.6834**	**0.7697**	**0.6204**
**N(4)**	**1.7946**	**1.0166**	**-0.4402**
**C(5)**	**2.1317**	**0.3020**	**-1.6060**
**C(6)**	**2.6534**	**-2.0632**	**-1.0361**
**C(7)**	**3.5573**	**-2.9725**	**-0.1946**
**C(8)**	**3.8225**	**-2.3410**	**1.1792**
**C(9)**	**4.5398**	**-0.9917**	**1.0338**
**C(10)**	**0.6217**	**1.8218**	**-0.3145**
**O(11)**	**1.5633**	**0.4077**	**-2.6730**
**O(12)**	**2.5534**	**1.1916**	**1.7505**
**C(13)**	**-0.4826**	**1.3110**	**0.3888**
**C(14)**	**-1.6461**	**2.0875**	**0.5287**
**C(15)**	**-1.6775**	**3.3642**	**-0.0409**
**C(16)**	**-0.5849**	**3.8706**	**-0.7460**
**C(17)**	**0.5674**	**3.0929**	**-0.8805**
**O(18)**	**-0.3835**	**0.0456**	**0.8902**
**C(19)**	**-1.3966**	**-0.2982**	**1.8730**
**N(20)**	**-2.7228**	**0.0579**	**1.4618**
**C(21)**	**-2.8215**	**1.5132**	**1.3007**
**C(22)**	**-3.3497**	**-0.7898**	**0.4959**
**C(23)**	**-3.6931**	**-0.3577**	**-0.7939**
**C(24)**	**-4.3370**	**-1.2256**	**-1.6815**

C(25)	-4.6302	-2.5380	-1.3080
C(26)	-4.2822	-2.9757	-0.0254
C(27)	-3.6598	-2.1085	0.8712
H(28)	3.9954	-0.6830	-2.0579
H(29)	4.5837	0.7349	-0.2425
H(30)	2.4444	-2.4991	-2.0191
H(31)	1.6854	-1.9670	-0.5247
H(32)	3.0793	-3.9539	-0.0809
H(33)	4.5121	-3.1448	-0.7146
H(34)	4.4302	-3.0100	1.8017
H(35)	2.8671	-2.2027	1.7058
H(36)	4.6298	-0.4892	2.0037
H(37)	5.5616	-1.1744	0.6733
H(38)	-2.5792	3.9655	0.0632
H(39)	-0.6291	4.8608	-1.1895
H(40)	1.4305	3.4638	-1.4248
H(41)	-1.2940	-1.3732	2.0081
H(42)	-1.1468	0.2303	2.8006
H(43)	-3.7710	1.7558	0.8164
H(44)	-2.8616	1.9557	2.3070
H(45)	-3.4394	0.6451	-1.1226
H(46)	-4.5915	-0.8720	-2.6775
H(47)	-5.1249	-3.2106	-2.0033
H(48)	-4.5156	-3.9905	0.2871
H(49)	-3.4324	-2.4405	1.8811

***o*HHI-a-B**

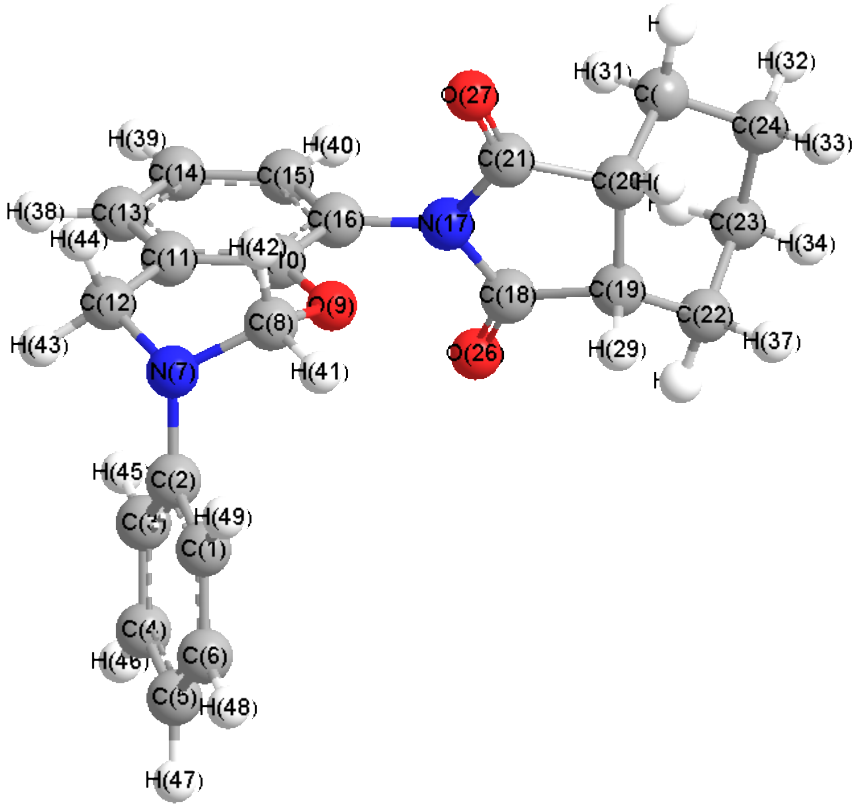

C(1)	-4.3501	-1.5920	1.1447
C(2)	-3.8088	-0.5098	0.4293
C(3)	-3.8951	-0.5170	-0.9710
C(4)	-4.5150	-1.5803	-1.6351
C(5)	-5.0389	-2.6584	-0.9202
C(6)	-4.9475	-2.6593	0.4760
N(7)	-3.2208	0.5631	1.1692
C(8)	-2.0341	0.2625	1.9155
O(9)	-0.8472	0.1660	1.0837
C(10)	-0.6929	1.1843	0.1893
C(11)	-1.7439	2.0533	-0.1521
C(12)	-3.0892	1.8779	0.5310
C(13)	-1.5111	3.0527	-1.1020
C(14)	-0.2657	3.1938	-1.7157
C(15)	0.7735	2.3269	-1.3704
C(16)	0.5643	1.3295	-0.4217
N(17)	1.6169	0.4374	-0.0525
C(18)	2.0330	-0.6514	-0.8405
C(19)	2.9415	-1.5163	0.0333
C(20)	3.3975	-0.5467	1.1481
C(21)	2.2910	0.5016	1.1803
C(22)	4.0338	-2.2976	-0.7122
C(23)	5.2977	-1.4716	-0.9896

**C(24)**	**5.8026**	**-0.7925**	**0.2912**
**C(25)**	**4.7415**	**0.1683**	**0.8417**
**O(26)**	**1.6749**	**-0.8620**	**-1.9804**
**O(27)**	**2.0588**	**1.3069**	**2.0581**
**H(28)**	**3.4622**	**-1.0389**	**2.1239**
**H(29)**	**2.2469**	**-2.2438**	**0.4807**
**H(30)**	**5.0848**	**0.6720**	**1.7520**
**H(31)**	**4.5720**	**0.9581**	**0.0954**
**H(32)**	**6.7283**	**-0.2372**	**0.0933**
**H(33)**	**6.0479**	**-1.5567**	**1.0445**
**H(34)**	**6.0724**	**-2.1234**	**-1.4125**
**H(35)**	**5.0860**	**-0.7042**	**-1.7487**
**H(36)**	**3.6096**	**-2.6894**	**-1.6439**
**H(37)**	**4.3102**	**-3.1659**	**-0.0983**
**H(38)**	**-2.3250**	**3.7242**	**-1.3706**
**H(39)**	**-0.1043**	**3.9701**	**-2.4577**
**H(40)**	**1.7515**	**2.4164**	**-1.8335**
**H(41)**	**-2.1037**	**-0.7060**	**2.4069**
**H(42)**	**-1.8551**	**1.0543**	**2.6524**
**H(43)**	**-3.9134**	**2.0179**	**-0.1734**
**H(44)**	**-3.2145**	**2.6399**	**1.3143**
**H(45)**	**-3.4594**	**0.2894**	**-1.5522**
**H(46)**	**-4.5686**	**-1.5680**	**-2.7208**
**H(47)**	**-5.5140**	**-3.4853**	**-1.4409**
**H(48)**	**-5.3624**	**-3.4845**	**1.0497**
**H(49)**	**-4.3220**	**-1.5774**	**2.2313**

*o*HTI-a-A

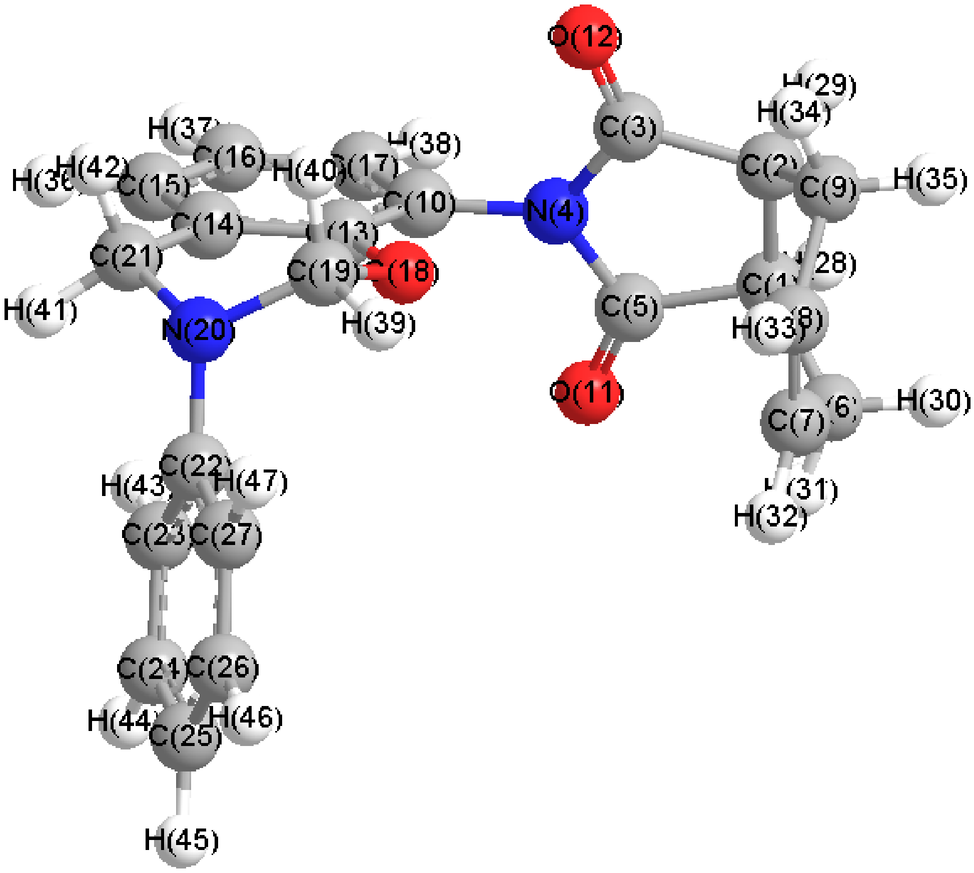

C(1)	-3.5548	-0.8007	1.2147
C(2)	-3.8741	-0.3954	-0.2445
C(3)	-2.8009	0.6314	-0.6122
N(4)	-1.9628	0.8179	0.4954
C(5)	-2.3045	0.0017	1.5820
C(6)	-3.3204	-2.3160	1.4387
C(7)	-2.5446	-2.9321	0.2998
C(8)	-2.8113	-2.5799	-0.9641
C(9)	-3.8909	-1.5673	-1.2598
C(10)	-0.8359	1.6967	0.4887
O(11)	-1.7063	-0.0349	2.6376
O(12)	-2.6910	1.2147	-1.6708
C(13)	0.2848	1.3700	-0.2936
C(14)	1.3976	2.2297	-0.3168
C(15)	1.3636	3.3998	0.4473
C(16)	0.2548	3.7214	1.2317
C(17)	-0.8468	2.8638	1.2487
O(18)	0.2544	0.1960	-0.9843
C(19)	1.2733	0.0643	-2.0098
N(20)	2.5812	0.4373	-1.5550

**C(21)**	**2.5915**	**1.8553**	**-1.1779**
**C(22)**	**3.2733**	**-0.5035**	**-0.7310**
**C(23)**	**3.6208**	**-0.2426**	**0.6030**
**C(24)**	**4.3318**	**-1.1902**	**1.3463**
**C(25)**	**4.6894**	**-2.4157**	**0.7827**
**C(26)**	**4.3376**	**-2.6836**	**-0.5448**
**C(27)**	**3.6481**	**-1.7344**	**-1.2975**
**H(28)**	**-4.3446**	**-0.4665**	**1.8986**
**H(29)**	**-4.8339**	**0.1311**	**-0.3086**
**H(30)**	**-4.3013**	**-2.8066**	**1.5304**
**H(31)**	**-2.8102**	**-2.4555**	**2.3969**
**H(32)**	**-1.7710**	**-3.6623**	**0.5276**
**H(33)**	**-2.2621**	**-3.0170**	**-1.7955**
**H(34)**	**-3.7966**	**-1.1584**	**-2.2708**
**H(35)**	**-4.8780**	**-2.0513**	**-1.2087**
**H(36)**	**2.2266**	**4.0636**	**0.4334**
**H(37)**	**0.2474**	**4.6301**	**1.8260**
**H(38)**	**-1.7212**	**3.0925**	**1.8506**
**H(39)**	**1.2336**	**-0.9833**	**-2.3016**
**H(40)**	**0.9809**	**0.7071**	**-2.8485**
**H(41)**	**3.5332**	**2.0848**	**-0.6724**
**H(42)**	**2.5837**	**2.4444**	**-2.1068**
**H(43)**	**3.3187**	**0.6854**	**1.0778**
**H(44)**	**4.5883**	**-0.9692**	**2.3794**
**H(45)**	**5.2363**	**-3.1514**	**1.3660**
**H(46)**	**4.6200**	**-3.6275**	**-1.0046**
**H(47)**	**3.4173**	**-1.9308**	**-2.3415**

*o*HTI-a-B

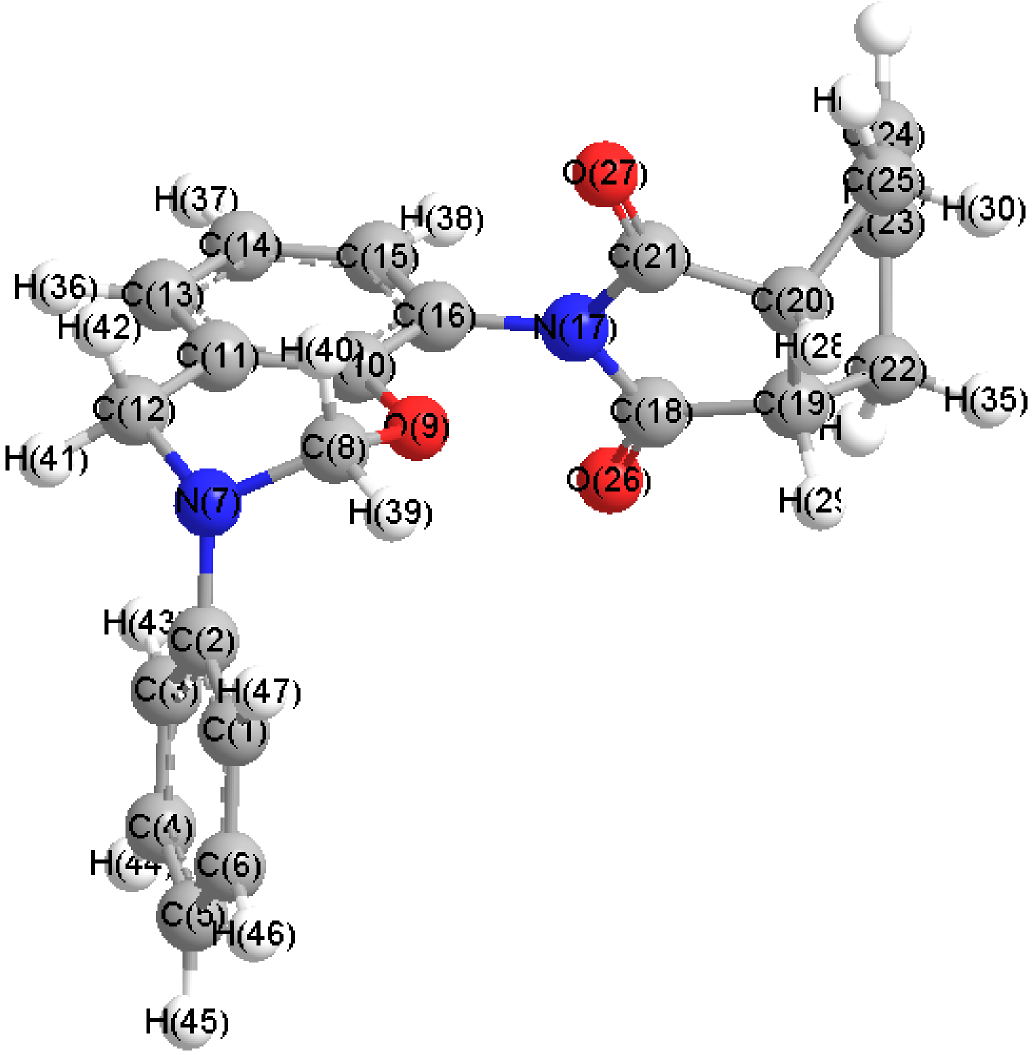

C(1)	0.0747	3.8567	-0.8803
C(2)	0.5355	2.5289	-0.8516
C(3)	0.9008	1.9164	-2.0598
C(4)	0.8121	2.6213	-3.2644
C(5)	0.3413	3.9349	-3.2883
C(6)	-0.0321	4.5468	-2.0867
N(7)	0.6383	1.8762	0.4161
C(8)	-0.5852	1.6356	1.1237
O(9)	-1.3392	0.5136	0.5920
C(10)	-0.6032	-0.6120	0.3638
C(11)	0.8013	-0.5953	0.3051
C(12)	1.5275	0.7163	0.5485
C(13)	1.4790	-1.7875	0.0326
C(14)	0.7884	-2.9803	-0.1868
C(15)	-0.6066	-2.9898	-0.1249
C(16)	-1.2995	-1.8141	0.1523

**N(17)**	**-2.7257**	**-1.8062**	**0.2263**
**C(18)**	**-3.5646**	**-1.9663**	**-0.8839**
**C(19)**	**-5.0156**	**-1.8097**	**-0.4246**
**C(20)**	**-4.9269**	**-1.5695**	**1.1020**
**C(21)**	**-3.4314**	**-1.6112**	**1.4209**
**C(22)**	**-5.8635**	**-3.0368**	**-0.8451**
**C(23)**	**-5.6603**	**-4.2004**	**0.0952**
**C(24)**	**-5.5845**	**-3.9918**	**1.4163**
**C(25)**	**-5.7019**	**-2.5922**	**1.9704**
**O(26)**	**-3.1847**	**-2.1912**	**-2.0148**
**O(27)**	**-2.9218**	**-1.5034**	**2.5171**
**H(28)**	**-5.2665**	**-0.5599**	**1.3630**
**H(29)**	**-5.4028**	**-0.9241**	**-0.9425**
**H(30)**	**-6.7602**	**-2.2915**	**1.9979**
**H(31)**	**-5.3328**	**-2.5325**	**2.9991**
**H(32)**	**-5.4466**	**-4.8200**	**2.1084**
**H(33)**	**-5.5869**	**-5.2037**	**-0.3196**
**H(34)**	**-5.6128**	**-3.3015**	**-1.8771**
**H(35)**	**-6.9234**	**-2.7405**	**-0.8456**
**H(36)**	**2.5666**	**-1.7757**	**-0.0177**
**H(37)**	**1.3300**	**-3.8958**	**-0.4054**
**H(38)**	**-1.1667**	**-3.9049**	**-0.2900**
**H(39)**	**-1.2650**	**2.4821**	**1.0494**
**H(40)**	**-0.3628**	**1.4134**	**2.1740**
**H(41)**	**2.3737**	**0.8344**	**-0.1334**
**H(42)**	**1.9422**	**0.7322**	**1.5671**
**H(43)**	**1.2312**	**0.8826**	**-2.0735**
**H(44)**	**1.0975**	**2.1271**	**-4.1898**
**H(45)**	**0.2682**	**4.4765**	**-4.2274**
**H(46)**	**-0.3880**	**5.5742**	**-2.0849**
**H(47)**	**-0.1744**	**4.3528**	**0.0543**

*o*PP-a

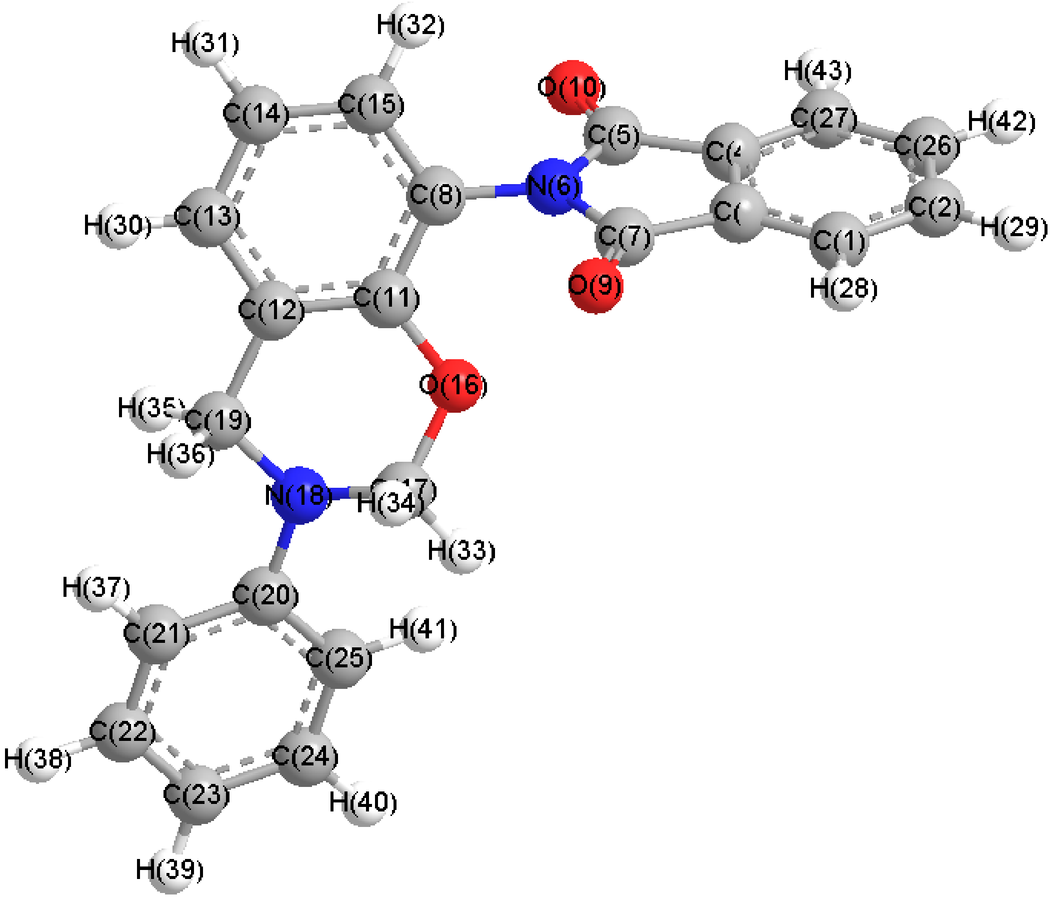

C(1)	4.7973	-1.6546	1.3526
C(2)	5.7853	-2.3506	0.6406
C(3)	4.0113	-0.7556	0.6436
C(4)	4.1963	-0.5456	-0.7244
C(5)	3.2063	0.4714	-1.1854
N(6)	2.4733	0.8374	-0.0324
C(7)	2.9003	0.1234	1.1116
C(8)	1.3653	1.7344	-0.0384
O(9)	2.4443	0.2414	2.2296
O(10)	3.0403	0.9204	-2.3014
C(11)	0.0673	1.2164	0.1176
C(12)	-1.0407	2.0744	0.1276
C(13)	-0.8257	3.4494	-0.0184
C(14)	0.4583	3.9684	-0.1844
C(15)	1.5563	3.1044	-0.1944
O(16)	-0.0647	-0.1366	0.2436
C(17)	-1.3177	-0.5346	0.8016
N(18)	-2.4167	0.0664	0.0836
C(19)	-2.4447	1.5124	0.2606
C(20)	-3.6257	-0.6456	-0.0654
C(21)	-4.8737	-0.0316	0.1436

**C(22)**	**-6.0607**	**-0.7436**	**-0.0544**
**C(23)**	**-6.0347**	**-2.0816**	**-0.4474**
**C(24)**	**-4.7957**	**-2.6986**	**-0.6564**
**C(25)**	**-3.6087**	**-1.9946**	**-0.4784**
**C(26)**	**5.9693**	**-2.1406**	**-0.7344**
**C(27)**	**5.1723**	**-1.2276**	**-1.4394**
**H(28)**	**4.6473**	**-1.8096**	**2.4166**
**H(29)**	**6.4183**	**-3.0636**	**1.1606**
**H(30)**	**-1.6827**	**4.1194**	**-0.0164**
**H(31)**	**0.6053**	**5.0374**	**-0.3064**
**H(32)**	**2.5653**	**3.4854**	**-0.3184**
**H(33)**	**-1.3487**	**-1.6186**	**0.7326**
**H(34)**	**-1.3167**	**-0.2316**	**1.8636**
**H(35)**	**-3.0837**	**1.9444**	**-0.5164**
**H(36)**	**-2.8767**	**1.8014**	**1.2386**
**H(37)**	**-4.9297**	**1.0024**	**0.4676**
**H(38)**	**-7.0117**	**-0.2426**	**0.1126**
**H(39)**	**-6.9587**	**-2.6336**	**-0.5954**
**H(40)**	**-4.7517**	**-3.7356**	**-0.9804**
**H(41)**	**-2.6627**	**-2.4826**	**-0.6954**
**H(42)**	**6.7433**	**-2.6946**	**-1.2594**
**H(43)**	**5.3073**	**-1.0566**	**-2.5034**

References

(1) Low, B. T.; Xiao, Y.; Tai, S. C.; Ye, L. Simultaneous occurrence of chemical grafting, cross-linking, and etching on the surface of polyimide membranes and their impact on H_2/CO_2 separation. *Macromolecules* **2008**, *41*, 1297-1309.

(2) Dunkers, J.; Ishida, H. Vibrational assignments of 3-alkyl-3, 4-dihydro-6-methyl-2H-1,3-benzoxazines in the Fingerprint Region. *Spectrochim. Acta.* **1995**, *51A*, 1061-1074.

(3) Han, L.; Iguchi, D.; Gil, P.; Heyl, T. R.; Sedwick, V. M.; Arza, C. R.; Ohashi, S.; Lacks, D. J.; Ishida, H. Oxazine ring-related vibrational modes of benzoxazine monomers using fully aromatically substituted, deuterated, ^{15}N isotope exchanged, and oxazine-ring-substituted compounds and theoretical calculations. *J. Phys. Chem. A.* **2017**, *121*, 6269-6282.
